# IRE1 promotes neurodegeneration through autophagy-dependent neuron death in the *Drosophila* model of Parkinson’s disease

**DOI:** 10.1038/s41419-019-2039-6

**Published:** 2019-10-22

**Authors:** Cheng Yan, Jingqi Liu, Jiamei Gao, Ying Sun, Lei Zhang, Haiyun Song, Lei Xue, Lixing Zhan, Guanjun Gao, Zunji Ke, Yong Liu, Jingnan Liu

**Affiliations:** 1 Key Laboratory of Nutrition and Metabolism, Institute for Nutritional Sciences, Shanghai Institutes for Biological Sciences, Chinese Academy of Sciences, Shanghai, 200031 China; 2 School of Life Science and Technology, ShanghaiTech University, Shanghai, 201210 China; 3 The State Key Laboratory of Cell Biology, CAS Center for Excellence in Molecular Cell Science, Innovation Center for Cell Signaling Network, Institute of Biochemistry and Cell Biology, Shanghai Institutes for Biological Sciences, Chinese Academy of Sciences, Shanghai, 200031 China; 4 School of Life Science and Technology, Tongji University, Shanghai, 200092 China; 5 Department of Biochemistry, Basic Medical College, Shanghai University of Chinese Traditional Medicine, Shanghai, 201203 China; 6 Hubei Key Laboratory of Cell Homeostasis, College of Life Sciences; the Institute for Advanced Studies, Wuhan University, Wuhan, 430072 China; 7 Present Address: School of Medicine, Xinxiang University, Xinxiang, Henan 453003 China

**Keywords:** Macroautophagy, Cell death in the nervous system, Parkinson's disease

## Abstract

Abnormal aggregation of misfolded pathological proteins in neurons is a prominent feature of neurodegenerative disorders including Parkinson’s disease (PD). Perturbations of proteostasis at the endoplasmic reticulum (ER) triggers ER stress, activating the unfolded protein response (UPR). Chronic ER stress is thought to underlie the death of neurons during the neurodegenerative progression, but the precise mechanism by which the UPR pathways regulate neuronal cell fate remains incompletely understood. Here we report a critical neurodegenerative role for inositol-requiring enzyme 1 (IRE1), the evolutionarily conserved ER stress sensor, in a *Drosophila* model of PD. We found that IRE1 was hyperactivated upon accumulation of α-synuclein in the fly photoreceptor neurons. Ectopic overexpression of IRE1 was sufficient to trigger autophagy-dependent neuron death in an XBP1-independent, JNK-dependent manner. Furthermore, IRE1 was able to promote dopaminergic neuron loss, progressive locomotor impairment, and shorter lifespan, whereas blocking IRE1 or ATG7 expression remarkably ameliorated the progression of α-synuclein-caused Parkinson’s disease. These results provide in vivo evidence demonstrating that the IRE1 pathway drives PD progression through coupling ER stress to autophagy-dependent neuron death.

## Introduction

Neurodegenerative diseases share a prominent pathological feature of disturbed proteostasis, which is characterized by the accumulation and aggregation of specific misfolded proteins within the affected neurons^1^. These protein-misfolding disorders include Parkinson’s disease (PD), Alzheimer’s disease (AD), Huntington’s disease (HD), prion-related diseases, and amyotrophic lateral sclerosis (ALS)^2^. PD is the second most common neurodegenerative disease with the hallmark of aggregation of α-synuclein in Lewy bodies, which is believed to cause selective death of dopaminergic neurons in the substantia nigra pars compacta^2–4^. Despite that several mechanisms such as mitochondrial dysfunction, oxidative stress, and defective intracellular Ca^2+^ homeostasis have been implicated in dopaminergic (DA) neuron degeneration, no neuroprotective therapies are currently available owing to our limited understanding of whether a unifying mechanism is at work to drive the pathogenic progression of PD.

Emerging lines of evidence suggest a close association between chronic endoplasmic reticulum (ER) stress and neurodegenerative conditions, including PD^1,5–8^. Alpha-synuclein, the key neurotoxic protein involved in PD, accumulates within the ER both in animal models of α-synucleinopathy and in human PD patients^9,10^. Perturbations of proteostasis at the ER, i.e. an overload of unfolded or misfolded proteins, cause ER stress and activate the adaptive unfolded protein response (UPR)^1^. In mammals, the UPR program is governed by three evolutionarily conserved ER transmembrane signal transducers, inositol-requiring enzyme 1 (IRE1), protein kinase RNA-like ER kinase (PERK), and activating transcription factor 6 (ATF6)^11,12^. As a homeostatic mechanism, the three UPR pathways cooperate to mitigate ER stress; but when ER homeostasis cannot be restored, cell death ensues^11,12^. However, the exact mechanism linking chronic activation of the UPR pathways to neuronal cell death remains largely elusive.

IRE1 is the most ancient ER stress sensor that is highly conserved from yeast to fruit fly and to mammals. It possesses both Ser/Thr protein kinase and endoribonuclease (RNase) activities in its cytoplasmic portion^11,13^. Upon ER stress, IRE1 is activated through trans-autophosphorylation and dimerization/oligomerization^13^, initiating a key branch of the UPR through catalyzing the unconventional splicing of X-box binding protein 1 (Xbp1) mRNA to generate XBP1s, the active spliced form of this transcription factor. Many studies have shown that IRE1 has a pivotal part in cell fate decision under ER stress conditions^13,14^. Whereas IRE1 is thought to promote cell survival by XBP1s-mediated enhancement of the ER’s protein folding capacity, recent studies have indicated that IRE1 can control cell death through regulated IRE1-dependent decay (RIDD)^15^ of the mRNA encoding death receptor 5 (DR5)^16^ or through cleavage of certain microRNA regulators of apoptosis^17–19^. Notably, the exact role of IRE1-XBP1 pathway in linking chronic ER stress to neuronal cell death appears to depend upon the disease context^1^. For instance, Valdés et al. showed that XBP1s exerted neuroprotective actions against a PD-inducing neurotoxin and promotes the survival of nigral DA neurons^20^. Casas-tinto et al. reported that XBP1 could suppress amyloid-beta neurotoxicity in a *Drosophila* AD model^21^. Similarly, reduction of Xbp1 gene dosage was shown to accelerate retinal degeneration in a *Drosophila* model for autosomal dominant retinitis pigmentosa^22^. In contrast, Vidal et al. and Hetz et al. reported that XBP1 deficiency resulted in protection against neurodegeneration in the transgenic mouse models of both HD^23^ and ALS^24^, likely through enhancement of autophagy. Moreover, IRE1 was also suggested as a crucial mediator of ER stress-induced aggregation of mutant huntingtin via suppressing autophagy flux, thereby leading to its neuronal toxicity in HD^25^. Autophagy is a highly conserved catabolic process^26^ and plays critical roles in proteostasis, tissue homeostasis and cell survival through lysosomal degradation of aggregate-prone proteins and intracellular organelles such as mitochondria and ER. Deregulation of the autophagic response may contribute to the development of neurodegenerative diseases^27,28^. Interestingly, reported studies indicated that the IRE1-JNK pathway might mediate autophagy activation and thus rendered cells more resistant to ER stress^29,30^. However, while being a topic of debate, emerging evidence also indicated that overactive autophagy might act as a lethal mechanism leading to “autophagy-dependent cell death” under certain physiological and pathological conditions^31–39^. Given their concurrent activation in the neurodegenerative states, it is of great significance to decipher whether the interplay of the IRE1 pathway and autophagy underlies the pathogenic progression of PD and other neurodegenerative disorders.

Here we investigated whether the IRE1 pathway links chronic ER stress and autophagy to autophagy-dependent neuron death in vivo. We utilized the well-established PD model in the fruit fly *Drosophila melanogaster*^40^ via ectopically expressing human α-synuclein in the photoreceptor or DA neurons. We found that overexpression of wild type or missense mutant α-Syn in photoreceptor neurons induced the activation of IRE1. Chronic activation of IRE1 triggered strong autophagy and induced cell loss in photoreceptor neurons. Unexpectedly, inhibition of autophagy by knockdown of *Atg* genes, *Atg7* or *Atg8b*, did prevent IRE1-caused neuron death. The autophagy-dependent neuron death induced by IRE1 was mediated by JNK signaling in an XBP1-independent manner. Our data demonstrate that in response to the accumulation of neurotoxic proteins, the IRE1 pathway serves as an unanticipated critical proteostatic “rheostat” to trigger autophagy-dependent neuron death, thereby driving the onset and progression of neurodegeneration in PD.

## Results

### IRE1 activation is associated with α-synucleinopathy and promotes neuronal degeneration

As the most accessible organ of the nervous system, the fly eye is dispensable for life and has been widely used to model neurodegeneration^41^. We first tested whether the IRE1 pathway is activated upon α-synucleinopathy in the photoreceptor neurons of *Drosophila*. We specifically overexpressed the human wild-type (WT) or two missence mutant forms (A30P and A53T) of α-synuclein identified from familial PD^40,42^ in the photoreceptor neurons. In 1-day old adult flies, histology analyses of the tangential sections showed normal retinal morphology and architecture, with well-organized R1-R7 photoreceptors observed in each ommatidium (Fig. S1a). By contrast, at 30 days of age, overt retinal degeneration, as manifested by the apparent loss of photoreceptor neurons along with vacuole formation, was observed in the GMR-Gal4 > α-Syn^WT^, GMR-Gal4 > α-Syn^A30P^ and GMR-Gal4 > α-Syn^A53T^ flies when compared to the GMR-Gal4 > + control line (Fig. S1a). Quantification of photoreceptor loss in tangential sections as analyzed by the percentage of intact ommatidia at each time point showed that ectopic expression of α-Syn^WT^, α-Syn^A30P^ or α-Syn^A53T^ resulted in progressive photoreceptor degeneration, with 45%, 55% and 47% of intact ommotidia observed, respectively, in GMR Gal4 > α-Syn^WT^, GMR Gal4 > α-Syn^A30P^ and GMR Gal4 > α-Syn^A53T^ flies relative to 94% in GMR Gal4 > + flies (Fig. S1b). No significant differences were found between flies expressing the WT and mutant α-synulein proteins. Subsequent immunoblot analyses of fly eyes revealed that this α-synucleinopathy was accompanied by elevated phosphorylation of IRE1 at Ser^703^ (Fig. S1c), a conserved site that corresponds to Ser^724^ within the activation loop of the kinase domain of murine IRE1α^43^. Notably, a higher extent of increase in IRE1 phosphorylation was detected in GMR-Gal4 > α-Sy n^A30P^ and GMR-Gal4 > α-Syn^A53T^ eyes than that in GMR-Gal4 > α-Syn^WT^ counterparts (Fig. S1c). In accordance, elevations in *Xbp1* mRNA splicing were also detected in fly eyes expressing α-synulein proteins (Fig. S1d), which indicates more severe ER stress induced by α-synuclein proteins. Moreover, elevations of IRE1 phosphorylation were paralleled by increased phosphorylation levels of c-Jun N-terminal kinase (JNK) (Fig. S1c), suggesting that α-synuclein-induced activation of IRE1 might be coupled to the JNK pathway during neuronal degeneration.

**Fig. 1:**
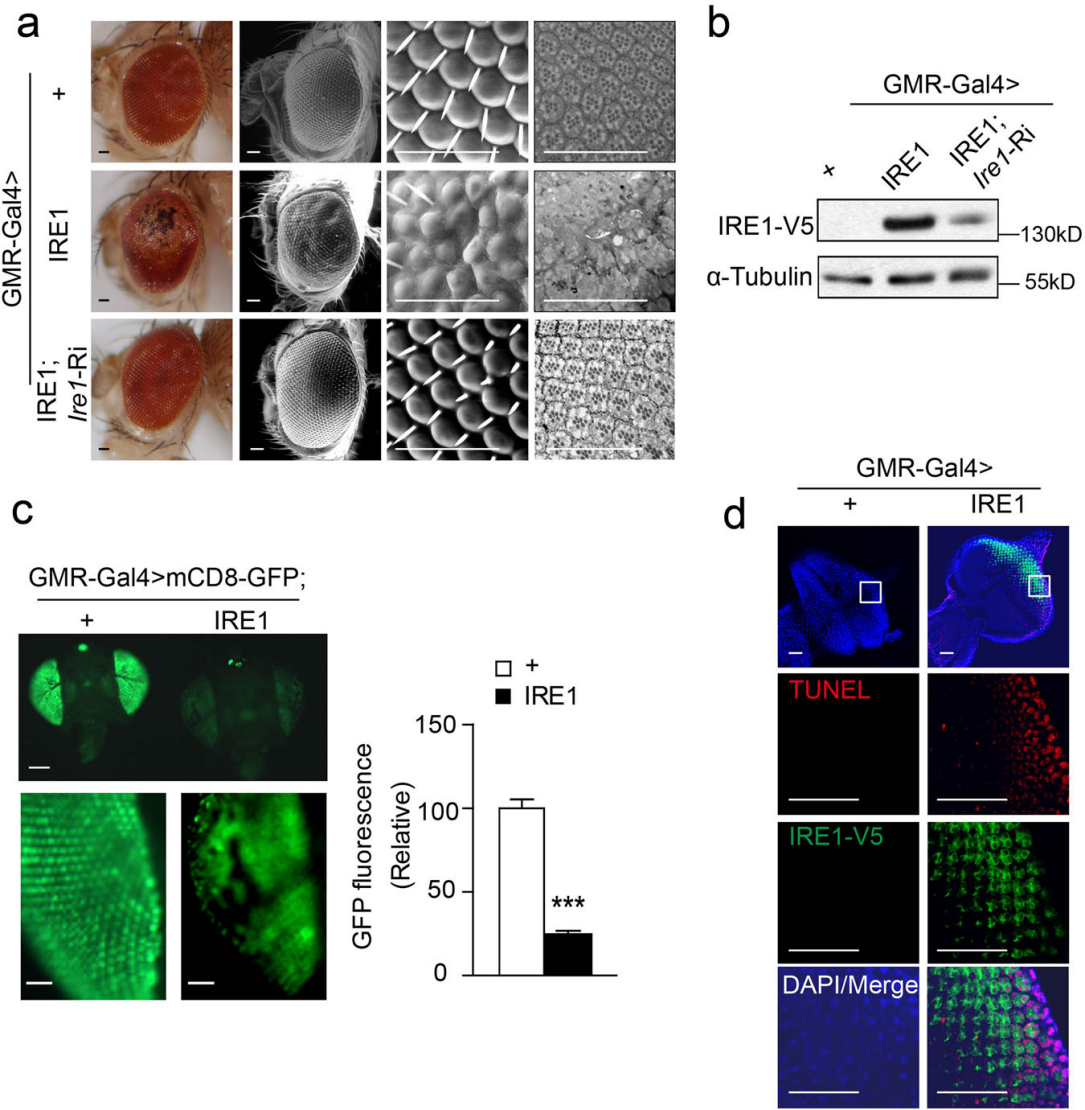
IRE1 drives neuronal death in *Drosophila*. **a**–**d** Ectopic overexpression of IRE1 is sufficient to induce neuronal cell death. **a** Representative light microscopy (left) and scanning electron microscopy (SEM) (middle) images of external adult fly eyes, along with tangential sections of adult eyes stained with toluidine blue (right) for the indicated lines (*n* = 3–5 flies/genotype). Scale bar represents 50 µm. **b** Immunoblot analysis of IRE1 expression from the head lysates of adult GMR-Gal4 > + versus GMR-Gal4 > IRE1 and GMR-Gal4 > IRE1; *Ire1*-Ri flies (*n* = 30 flies/genotype; representative of two independent experiments). Anti-V5 antibody was used. **c** Representative fluorescent mi**c**roscopy images of heads of adult GMR-Gal4 > mCD8-GFP versus GMR-Gal4 > mCD8-GFP; IRE1 flies. Shown at bottom are the enlarged images of the individual ommatidium. Scale bar represents 50 µm. Fluorescence signals were quantified from three independent experiments and are presented as mean ± s.e.m. (*n* = 10 flies/genotype). ***P *<* 0.001 by Student’s *t* test. **d** Cell death analysis of eye discs from 3rd instar larvae of the indicated genotypes. Shown are representative images of TUNEL labeling along with IRE1 immunofluorescent staining with anti-V5 antibody with the enlarged regions indicated (*n* = 20 flies/genotype). Scale bar represents 30 µm

To determine if IRE1 is involved in such α-synucleinopathy, we inhibited its expression by RNAi. We found that IRE1 deficiency markedly rescued α-Syn-evoked retinal degeneration, as shown by 70%, 72%, and 62% of intact ommotidia, respectively, in retina from GMR-Gal4 > α-Syn^WT^; *Ire1*-Ri, GMR-Gal4 > α-Syn^A30P^; *Ire1*-Ri, and GMR-Gal4 > α-Syn^A53T^; *Ire1*-Ri flies at 30 days of age (Fig. S1a, b). Next, we wondered if hyperactivation of IRE1 is sufficient to mediate α-synuclein’s neurotoxic effects. To test this idea, we generated transgenic flies with specific overexpression of V5-tagged *Drosophila* IRE1 in the photoreceptor neurons. Remarkably, overexpression of IRE1 caused large anomalies to the external eyes in comparison to those of GMR-Gal4 > + flies, and scanning electron microscopy (SEM) analysis revealed a glassy eye surface characterized by ommatidial disruption and loss of interommatidial bristles (Fig. 1a). Histological examination of the tangential sections also showed massive loss of photoreceptor neurons in IRE1-expressing eyes (Fig. 1a). To exclude the possible non-specific effects of IRE1 transgene insertion, we knocked down the expression of IRE1 by RNAi in the eyes of IRE1-expressing flies, and confirmed that the retinal neuron loss indeed resulted from IRE1 overexpression (Fig. 1a, b). We analyzed the mRNA abundance of *Crc* (the *Drosophila* homolog of ATF4, the downstream marker of the PERK pathway), *Atf6* as well as *PEK* (the *Drosophila* homolog of PERK) in the adult head of GMR-Gal4 > IRE1 flies. No significant changes were observed in the expression of these UPR signaling genes (Fig S2), indicating that neither the PERK nor the ATF6 pathway was likely to have a critical role in IRE1-induced neuron loss.

To further quantitatively determine the extent of IRE1-induced neuron loss, we used the mCD8-GFP reporter system^44^ and found that IRE1 overexpression resulted in ~75% loss of the retinal neurons in GMR-Gal4 > mCD8-GFP; IRE1 flies (Fig. 1c). In addition, TUNEL analyses showed prominent IRE1-induced cell death in the eye imaginal discs of GMR-Gal4 > IRE1 larvae (Fig. 1d). These data demonstrated that IRE1 was sufficient to instigate neuronal cell death in *Drosophila*, which might mediate α-synuclein induction of neuronal degeneration.

### IRE1 induces autophagy-dependent neuron death

**Fig. 2:**
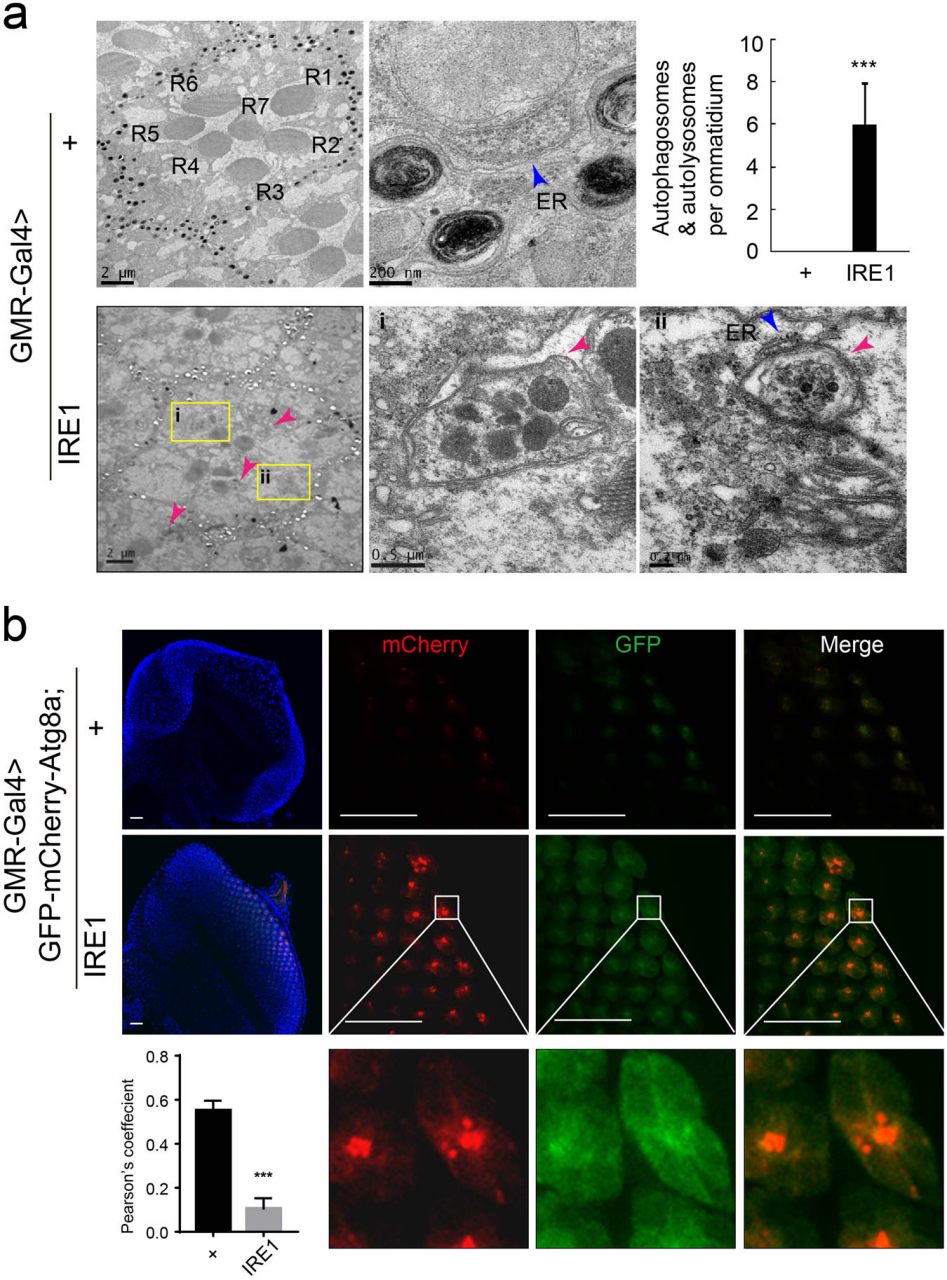
IRE1 overexpression results in enhanced autophagy. **a** Transmission electron microscopy (TEM) analysis of the tangential section of an ommatidium from adult GMR-Gal4 > + versus GMR-Gal4 > IRE1 flies (*n* = 3–5 flies/genotype). Shown are representative micrographs with the scale bars indicated (2 µm, 0.2 µm, 0.5 µm, and 200 nm). Arrows indicate the autophagosomes/autolysosomes (pink), ER endoplasmic reticulum (blue). The quantitative data are shown in the right panel. Data are shown as mean ± s.e.m. (*n* = 3–5 flies/genotype; three independent experiments). ***P *<* 0.001 by Student’s *t* test. Scale bar represents as indicated. **b** Analysis of autophagy flux in the dual-tagged mCherry-GFP-Atg8a reporter line. Representative confocal micrographs of eye discs from 3rd instar larvae of GMR-Gal4 > GFP-mCherry-Atg8a versus GMR-Gal4 > GFP-mCherry-Atg8a;IRE1 flies with the enlarged regions indicated (*n* = 20 flies/genotype). Enlarged images showing the autophagosomes/autolysosomes visualized as the puncta are indicated in white boxes. Scale bar represents 25 µm

To determine the cellular characteristics of IRE1-induced neuron death, we analyzed the tangential sections of fly eyes by transmission electron microscopy (TEM). GMR-Gal4 > + control flies had well-organized photoreceptors in each ommatidium, along with normal structural appearance of the ER and mitochondria (Fig. 2a). In contrast, GMR-Gal4 > IRE1 flies showed severe derangement or loss of the photoreceptor neurons, and TEM analyses revealed overt accumulation of autophagosomes/autolysosomes encircling mitochondria and amorphous structures which appeared to be the partially degraded cellular debris (Fig. 2a). This suggests that IRE1-induced neuronal loss was accompanied by activation of autophagy in the eyes of GMR-Gal4 > IRE1 flies. To further affirm the occurrence of autophagy, we used the dual-tagged GFP-mCherry-Atg8a reporter system^45^ to enable the detection of autophagy flux. Indeed, as compared to the GMR-Gal4 > + control, marked increases of red mCherry-Atg8a-derived puncta were observed in GMR-Gal4 > IRE1 eye discs due to the quenching of the GFP signal under autophagy-associated acidic conditions (Fig. 2b). Pearson’s coefficient is markedly decreased in GMR-Gal4 > IRE1 eye discs as compared to GMR-Gal4 > + control (Fig. 2b). To further affirm autophagy flux is functional, we examed the level of known autophagic substrate Ref(2)P, the fly homolog of the autophagy receptor p62, which degraded upon activation of autophagy flux^46,47^. Immunofluorescence staining of Ref(2)P on eye imaginal discs, as well as immune blot analysis, all showed dramatically decreased Ref(2)P in GMR-Gal4 > IRE1 flies relative to GMR-Gal4 > + control flies (Fig. S3a, b).

**Fig. 3:**
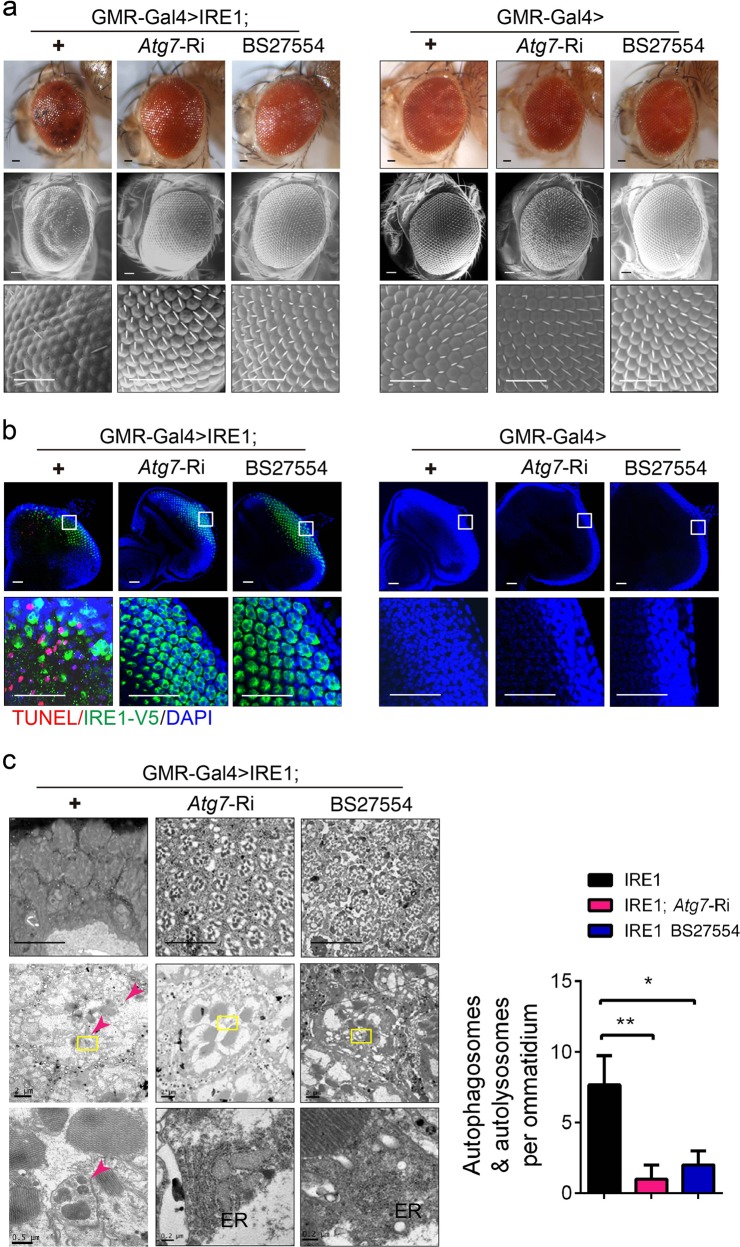
Autophagy is required for IRE1-induced neuron death. **a** Representative light microscopy (top) and SEM (middle and bottom) images of external eyes from adult GMR-Gal4 > IRE1, GMR-Gal4 > IRE1;*Atg7-*Ri and GMR-Gal4 > IRE1;*Atg8b-*Ri flies versus GMR-Gal4 > +, GMR*-*Gal4 > *Atg7-*Ri and GMR-Gal4 > *Atg8b-*Ri flies (*n* = 5–8 flies/genotype). Scale bar represents 50 µm. **b** Cell death analysis of larval eye discs of the indicated lines. Shown are representative images of TUNEL and DAPI staining along with IRE1 immunostaining with the enlarged regions indicated (*n* = 20 flies/genotype). Scale bar represents 30 µm. **c** Representative light microscopy images of tangential sections of adult eyes stained with toluidine blue (top panels) and TEM micrographs of the tangential section of an ommatidium (middle and bottom; Scale bars, 2 µm, 0.5 µm, and 0.2 µm) from the indicated lines (*n* = 3–5 flies/genotype). ER endoplasmic reticulum. Arrows indicate autophagosomes/autolysosomes, which were quantified from two independent experiments and are shown as mean ± s.e.m. (*n* = 3–5 flies/genotype). *P *<* 0.05, **P *<* 0.01 by Student’s *t* test. Scale bar represents 25 µm

ATG (autophagy-related) proteins are essential for forming the double-membrane autophagosomal vesicles and for the execution of autophagy, among which ATG7 is a key component of the core autophagy machinery^26,48^. Given that the constituent ATG molecules are evolutionarily conserved from *Drosophila* to mammals, we first examined their mRNA levels in IRE1-expressing eyes. Notably, although the expression of *Atg1*, *Atg9*, *Atg12*, *Atg6*, and *Atg8b* was considerably upregulated, no significant alteration in the expression of *Atg3*, *18a*, *Atg5*, or *Atg7* was detected in GMR-Gal4 > IRE1 eyes (Fig. S4). Next, to determine whether the enhancement of autophagy mediated IRE1-induced loss of photoreceptor neurons, we performed in vivo screening of a number of the available *Atg* RNAi fly lines (Fig. S5). Remarkably, in GMR-Gal4 > IRE1; *Atg7*-Ri or GMR-Gal4 > IRE1; *Atg8b*-Ri flies, knockdown by ~50% of the expression of *Atg7* or *Atg8b* (Fig. S6a, b) almost completely or partially rescued, respectively, IRE1-evoked retinal structural derangement (~70% of GMR-Gal4 > IRE1; *Atg8b*-Ri flies showed phenotypical ameliorations), with restored organization of ommatidia and interommatidial bristles observed (Fig. 3a); whereas knockdown of *Atg7* or *Atg8b* expression did not directly affect the morphology of GMR-Gal4 > *Atg7*-Ri or GMR-Gal4 > *Atg8b*-Ri eyes (Fig. 3a). TUNEL staining analyses of eye imaginal discs also revealed marked decreases in neuronal cell death (Fig. 3b), and TEM assessment showed significant reversal of IRE1-induced photoreceptor neuron loss, along with normalized structures of ER and mitochondria but no detectable accumulation of autophagosomes/autolysosomes in GMR-Gal4 > IRE1; *Atg7*-Ri or GMR-Gal4 > IRE1; *Atg8b*-Ri flies (Fig. 3c). Then we tested whether this autophagy-dependant cell death involves the action of caspases (cysteine aspartate-specific proteinases). Inhibition of caspases by overexpressing p35^49^, Dronc^DN^ ^50^ or homozygous mutation for *dronc*^*I29*^ null allele^51^ in GMR-Gal4 > IRE1; p35, GMR-Gal4 > IRE1; Dronc^DN^ or GMR-Gal4 > IRE1; *dronc*^*I29*^ flies showed no rescuing effects upon IRE1-evoked disorganization of ommatidia and bristles (Fig. S7), indicating that caspase-dependent apoptosis may not have a prominent role in IRE1-induced neuron loss. Thus, these results suggest that IRE1-initiated activation of autophagy critically contributed to IRE1 promotion of neuronal cell death.

### IRE1 drives neuronal cell death in an XBP1-independent fashion

**Fig. 4:**
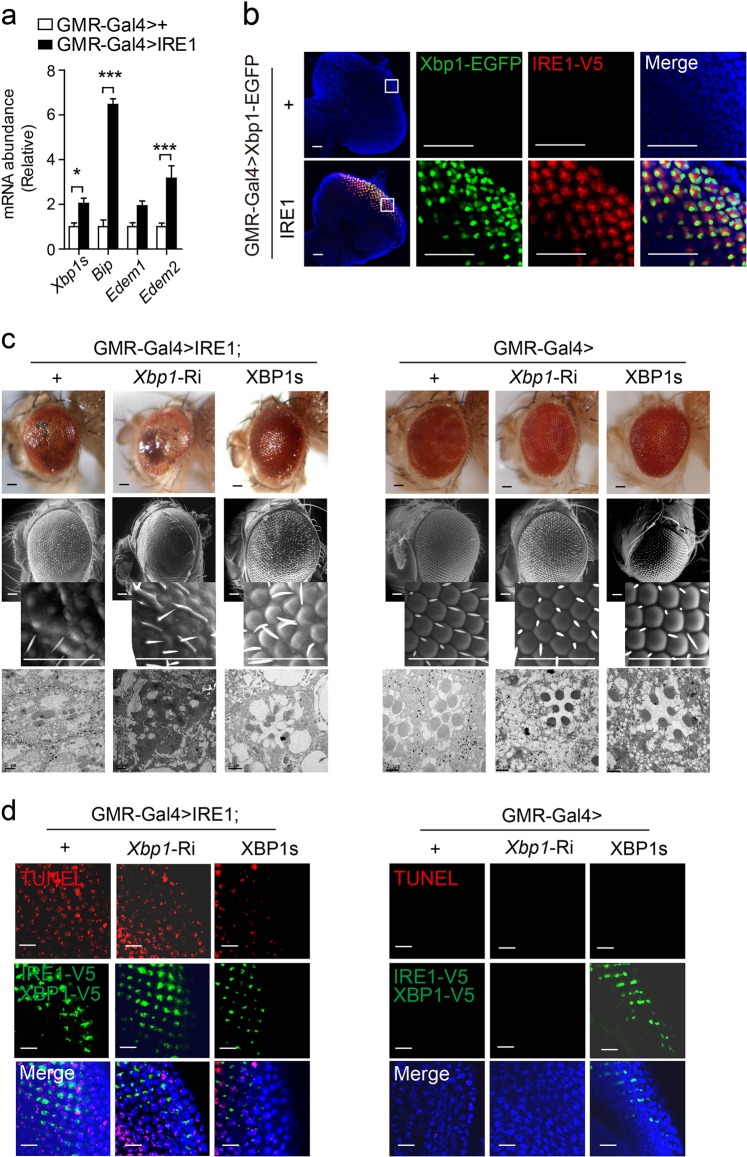
IRE1 induces neuron death in an XBP1-independent fashion. **a** Quantitative RT-PCR analysis of *Xbp1* mRNA splicing and the mRNA abundance of *Bip*, *Edem 1* and *Edem2* from the head lysates of adult GMR-Gal4 > + and GMR-Gal4 > IRE1 flies. Data are shown as mean ± s.e.m. (*n* = 30 flies/genotype; three independent experiments). *P *<* 0.05, ***P *<* 0.001 by Student’s *t* test. **b** Confocal microscopy analysis of eye discs from 3rd instar larvae of the GMR-Gal4 > Xbp1-EGFP versus GMR-Gal4 > Xbp1-EGFP;IRE1 line. Representative images are shown for *Xbp1* mRNA splicing-directed EGFP expression, along with IRE1 immunostaining with anti-V5 antibody and DAPI staining for eye discs with the enlarged regions indicated (*n* = 20 flies/genotype). Scale bar represents 30 µm. **c** Representative light microscopy (top), SEM (middle, with enlarged sections) and TEM (bottom) images of external eyes from adult GMR-Gal4 > IRE1, GMR-Gal4 > IRE1; *Xbp1-*Ri and GMR-Gal4 > IRE1; XBP1s flies versus GMR-Gal4 > +, GMR-Gal4 > *Xbp1-*Ri and GMR-Gal4 > XBP1s flies (*n* = 5–8 flies/genotype). Scale bar represents 50 µm. **d** Cell death analysis by TUNEL of eye discs from 3rd instar larvae of the indicated lines. Shown are representative images of TUNEL and DAPI staining along with IRE1 immunostaining (*n* = 20 flies/genotype). Scale bar represents 10 µm

Next, we asked if IRE1’s downstream effector XBP1 is involved. As anticipated, overexpressed IRE1 manifested an automatic activation state in GMR-Gal4 > IRE1 eyes, in which increased *Xbp1* mRNA splicing and elevated expression of XBP1s target genes, *Bip* and *Edem1/2*, were detected by quantitative RT-PCR (Fig. 4a). Further, when intercrossed to the Xbp1-EGFP reporter line in which EGFP expression is driven by *Xbp1* mRNA splicing^22^, prominent *Xbp1* mRNA splicing activity was detected in the eye discs of GMR-Gal4 > Xbp1-EGFP; IRE1 flies relative to the GMR-Gal4 > Xbp1-EGFP control line (Fig. 4b). Then, we examined if XBP1 could mediate IRE1-induced neuronal cell death. Surprisingly, knockdown by ~40% of the expression of *Xbp1* (Fig. S6c) had a more severe damaging effect upon the appearance of external eyes and the disorganization of ommatidia in GMR-Gal4 > IRE1; *Xbp1*-Ri flies, while overexpression of the spliced form of XBP1 (XBP1s, V5-tagged) appreciably attenuated these phenotypes in GMR-Gal4 > IRE1; XBP1s eyes (Fig. 4c). TUNEL analyses showed that suppression of *Xbp1* expression exerted no effect upon IRE1-induced cell death in the imaginal discs of GMR-Gal4 > IRE1; *Xbp1*-Ri larvae (Fig. 4d), whereas overexpression of XBP1s considerably reduced it in GMR-Gal4 > IRE1; XBP1s discs. Notably, neither knockdown of *Xbp1* expression nor XBP1s overexpression affected the fly eye morphology or cell viability in the absence of IRE1 overexpression (Fig. 4c, d). These results suggest that XBP1 does not mediate IRE1 promotion of neuronal loss; rather, XBP1s may function in a negative feedback loop to protect against IRE1-induced neuronal cell death. Therefore, IRE1 could drive neuronal cell death through a mechanism that is independent of XBP1 actions.

To investigate if RIDD has a role in mediating IRE1-induced photoreceptor neuron loss, we first evaluated changes in the mRNA levels of a selective set of RIDD targets under IRE1-overexpressing conditions, including the fatty acid transport protein (Fatp) that has been shown to be implicated in IRE1 regulation of photoreceptor differentiation and survival^52,53^. Quantitative RT-PCR analyses revealed significant decreases in the mRNA abundance of *Fatp*, *Cds and Indy* by ~50.8%, ~31.0%, and ~28.8%, respectively, in GMR-Gal4 > IRE1 eyes relative to their GMR-Gal4 > + controls (Fig. S7a). In addition, knockdown of the expression of IRE1 resulted in a trend of reversed increase in the mRNA levels of these RIDD targets (Fig. S8a). Despite that knockdown of the expression of *Fatp*, *Cds* or *Indy* did not affect the adult eye morphology (Fig. S8b), the possible contribution of these RIDD target genes remains to be dissected during autophagy-associated photoreceptor neuron loss in GMR-Gal4 > IRE1 flies.

### IRE1 promotes autophagy-dependent neuron death through JNK activation

**Fig. 5:**
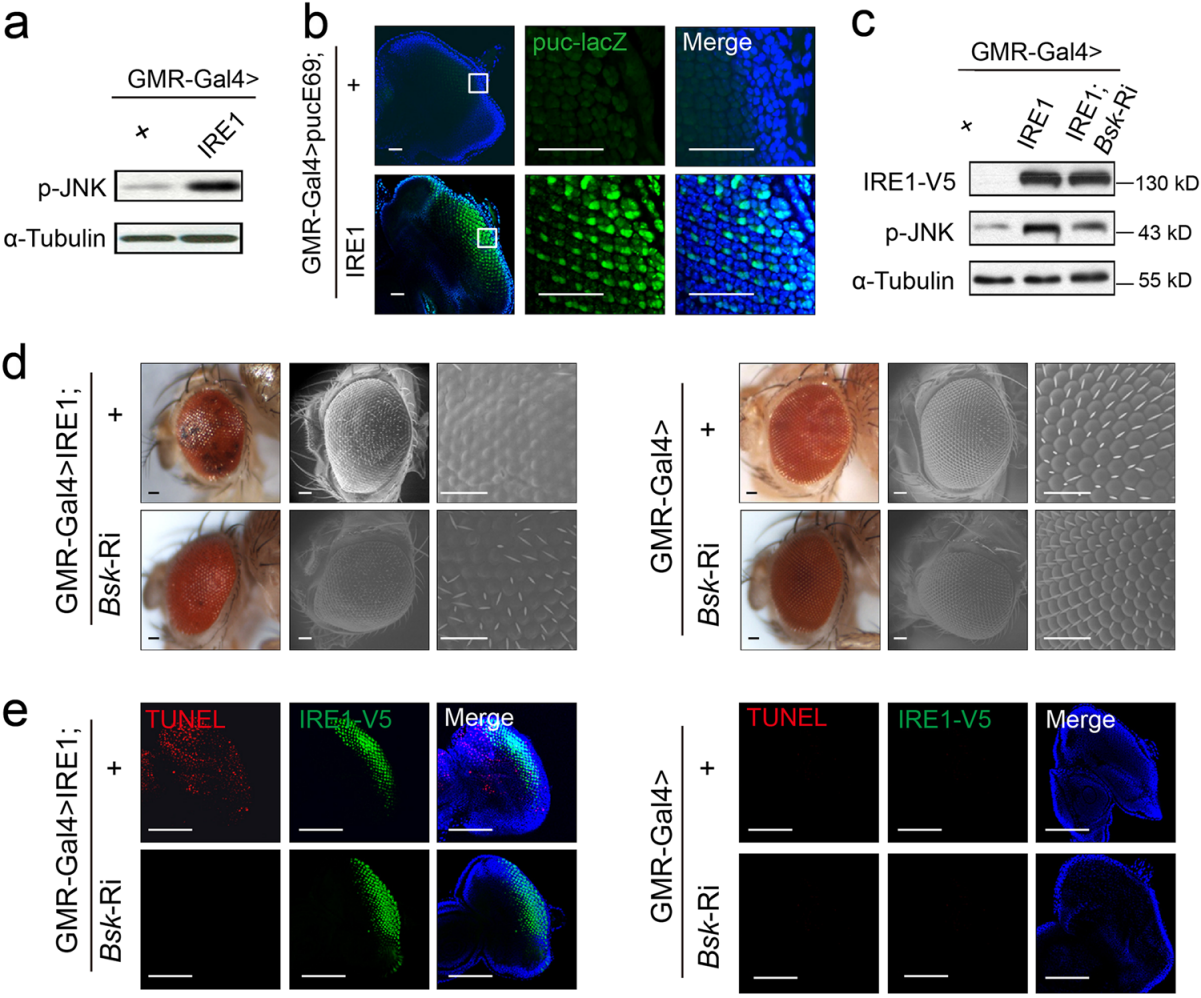
JNK mediates IRE1-induced autophagy-dependent neuron death. **a** Immunoblot analysis of the phosphorylation levels of JNK from the head lysates of adult GMR-Gal4 > + versus GMR-Gal4 > IRE1 flies (*n* = 30 flies/genotype; representative of three independent experiments). Scale bar represents 30 µm. **b** Confocal microscopy analysis of eye discs from GMR-Gal4 > puc^E69^ versus GMR-Gal4 > puc^E69^; IRE1 larvae. Shown are representative images for the JNK reporter *puc-lacZ* expression by immunostaining with anti-β-Gal antibody, along with DAPI staining (*n* = 20 flies/genotype). The enlarged regions are indicated. Scale bar represents 30 µm. **c** Immunoblot analysis of JNK phosphorylation in the adult head lysates of GMR-Gal4 > +, GMR-Gal4 > IRE1 and GMR-Gal4 > IRE1; *Bsk-*Ri lines (*n* = 30 flies/genotype; representative of three independent experiments). (**d**) Representative light microscopy (left panels) and SEM (middle and right panels) images of external eyes from adult GMR-Gal4 > IRE1 and GMR-Gal4 > IRE1; *Bsk-*Ri versus GMR-Gal4 > + and GMR-Gal4 > *Bsk-*Ri lines (*n* = 5–8 flies/genotype). Scale bar represents 50 µm. **e** Cell death analysis of larval eye discs from the indicated lines. Shown are representative images of TUNEL and DAPI staining along with IRE1 immunostaining (*n* = 20 flies/genotype). Scale bar represents 100 µm

IRE1 is known to be linked to JNK activation^54,55^. Given the association of α-synucleinopathy with phosphorylation activation of both IRE1 and JNK (Fig. S1b), we considered that IRE1 might promote autophagy-dependent neuron death through JNK signaling. Indeed, immunoblot analysis revealed increased JNK phosphorylation in the eyes of GMR-Gal4 > IRE1 flies (Fig. 5a). When intercrossed to the *puc-lacZ* reporter line for in vivo measurement of JNK activity^56^, markedly elevated activation of the IRE1-JNK pathway was observed in the imaginal discs of GMR-Gal4 > pucE69; IRE1 larvae (Fig. 5b). To determine the importance of JNK in IRE1 promotion of autophagy-dependent neuron death, we knocked down the expression of the JNK-encoding gene *Basket* (*Bsk*) in the eyes of GMR-Gal4 > IRE1; *Bsk*-Ri flies (Fig. 5c). While exerting no apparent effects upon the eyes of flies without IRE1 overexpression (Fig. 5d), knockdown of *Bsk* expression prominently alleviated IRE1-induced disruption of ommatidia (Fig. 5d) and significantly blocked IRE1-induced neuron death in the imaginal discs (Fig. 5e) in GMR-Gal4 > IRE1; *Bsk*-Ri flies. These data suggest that JNK acted as an important mediator in IRE1 promotion of autophagy-dependent neuron death.

### IRE1 causes Parkinsonian neurodegeneration through autophagy-dependent dopaminergic neuron loss

**Fig. 6:**
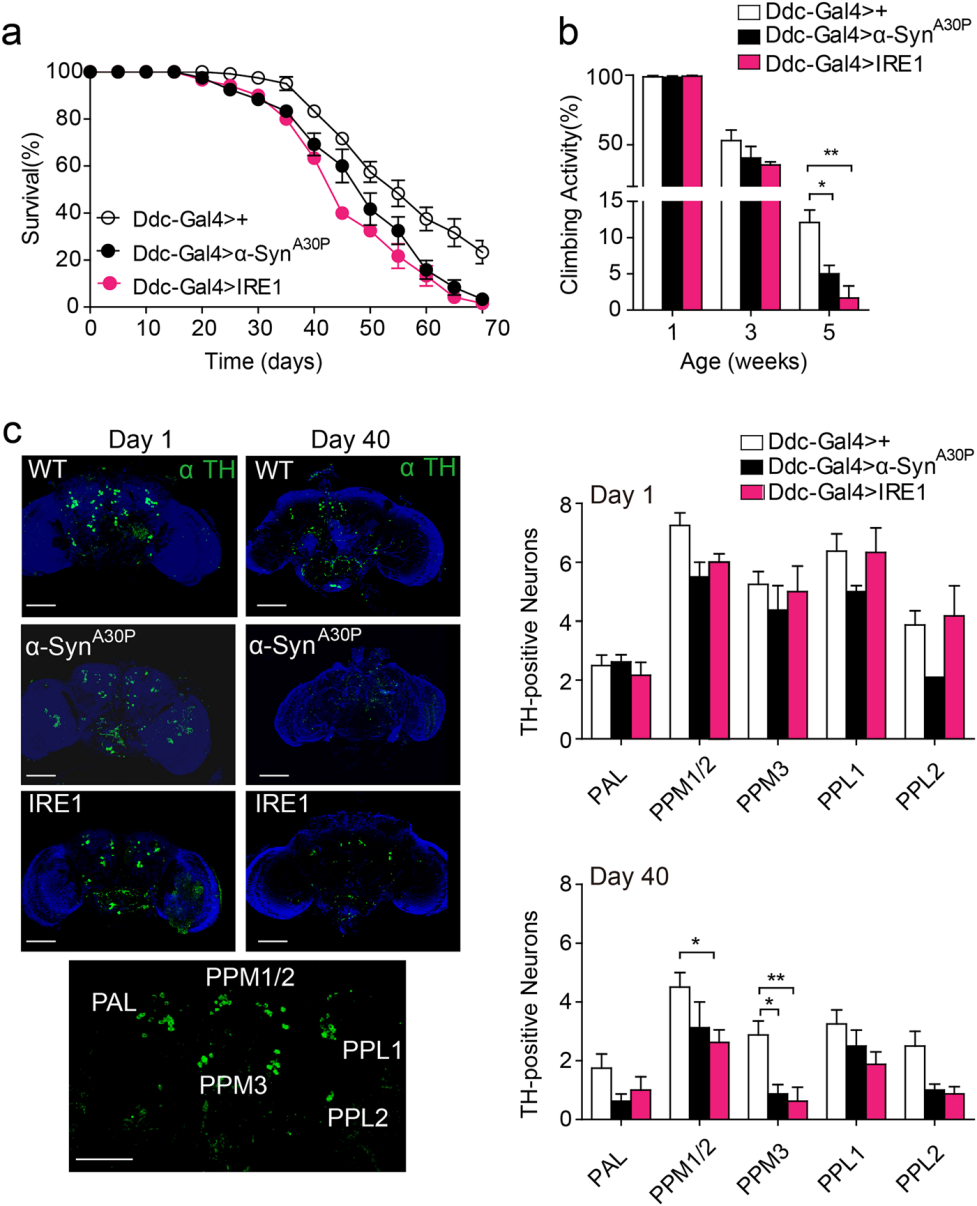
IRE1 in dopaminergic neurons promotes neurodegenerative progression. **a** Lifespan of Ddc-Gal4 > +, Ddc-Gal4 > α-Syn^A30P^, and Ddc-Gal4 > IRE1 lines (*n* = 90 flies/genotype). **b** Climbing ability of the indicated genotypes at 1, 3, or 5 weeks of age (*n* = 90 flies/genotype; three independent experiments). **c** Representative Z-stack confocal microscopy images of whole brains stained with the anti-tyrosine hydroxylase (TH) antibody for the indicated lines at 1 day or 40 days of age. Bottom: a representative image with the indicated TH-positive dopaminergic neuron clusters indicating the names, PAL protocerebral anterior lateral, PPM protocerebral posterior medial, PPL protocerebral posterior lateral. Dopaminergic neurons in the indicated clusters were quantified (*n* = 5 flies/genotype; two independent experiments). All data are shown as mean ± s.e.m. *P *<* 0.05, **P *<* 0.01 by one-way ANOVA. Scale bar represents 100 µm

To determine whether IRE1-induced neuron death underlies the neurodegenerative progression in the PD model, we first examined the effects of IRE1 or α-Syn^A30P^ overexpression upon DA neurons in the brain using the Ddc-Gal4 > driver. Remarkably, Ddc-Gal4 > IRE1 flies, in which IRE1 was overexpressed in their DA neurons, phenotypically mimicked Ddc-Gal4 > α-Syn^A30P^ flies, exhibiting similarly shorter lifespan (Fig. 6a) and age-dependent impairment in their climbing ability relative to the control Ddc-Gal4 > + line (Fig. 6b). Furthermore, assessment of the integrity of DA neurons showed that, at 1 day of age, IRE1 or α-Syn^A30P^ overexpression had no apparent effect upon the number of DA neurons in the five indicated clusters (Fig. 6c), suggesting that IRE1 overexpression did not disrupt the development of DA neurons. However, at 40 days of age, Ddc-Gal4 > IRE1 flies exhibited significant loss of DA neurons in some of the clusters, similar to the extent of DA neuron loss observed in Ddc-Gal4 > α-Syn^A30P^ flies (Fig. 6c). Therefore, chronic activation of IRE1 by its overexpression was sufficient to cause DA neuron degeneration.

**Fig. 7:**
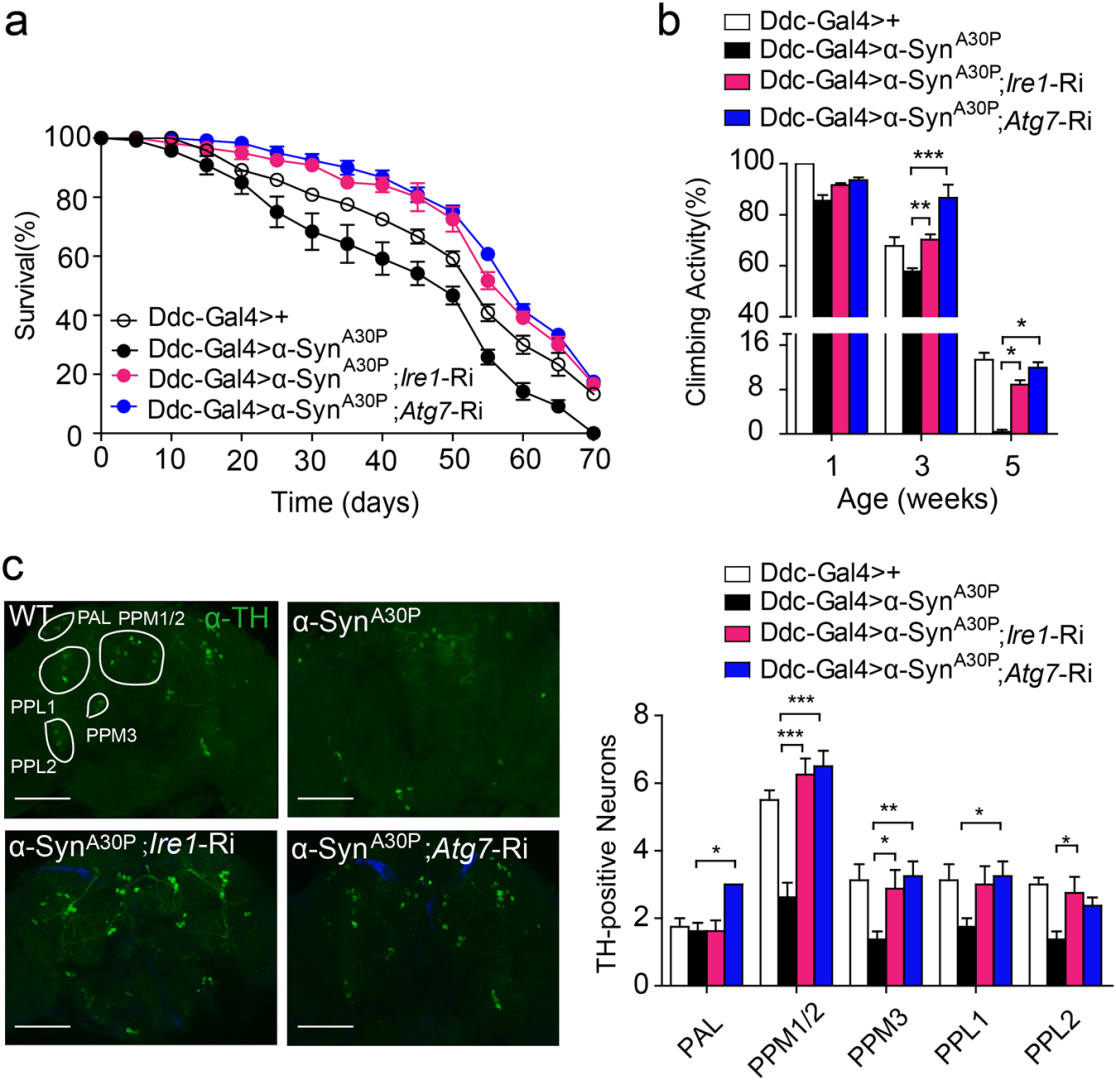
Knockdown of IRE1 or ATG7 expression reverses α-Synuclein-induced neurodegeneration. **a** Lifespan of Ddc-Gal4 > +, Ddc-Gal4 > α-Syn^A30P^, Ddc-Gal4 > α-Syn^A30P^; *Ire1*-Ri, and Ddc-Gal4 > α-Syn^A30P^; *Atg7*-Ri flies (*n* = 90 flies/genotype). **b** Climbing ability of the indicated genotype at 1, 3, or 5 weeks of age. Relative activities are shown as mean ± s.e.m. (*n* = 90 flies/genotype; two independent experiments). *P *<* 0.05, **P *<* 0.01, ***P *<* 0.001 by one-way ANOVA. **c** Representative Z-stack confocal images of whole brains stained with anti-TH antibody for the indicated lines at 40 days of age. TH-positive DA neurons of the indicated clusters were quantified. PAL protocerebral anterior lateral, PPM protocerebral posterior medial, PPL protocerebral posterior lateral. The data are shown as mean ± s.e.m. (*n* = 5 flies/genotype; two independent experiments). *P *<* 0.05, **P *<* 0.01, ***P *<* 0.001 by one-way ANOVA. Scale bar represents 100 µm

Then we determined if IRE1 or autophagy in DA neurons is essential in α-synuclein-induced neurodegeneration. Indeed, knockdown of the expression of *Ire1* or *Atg7* in Ddc-Gal4 > α-Syn^A30P^; *Ire1*-Ri or Ddc-Gal4 > α-Syn^A30P^; *Atg7*-Ri flies markedly rescued α-Syn^A30P^-evoked neurodegenerative phenotypes, with complete correction of their lifespan and locomotive activity observed (Fig. 7a, b). In addition, this phenotypical reversal was associated with significant suppression of α-Syn^A30P^-induced loss of DA neurons when examined at 40 days of age (Fig. 7c). Of interesting note, in the absence of α-Syn^A30P^ overexpression, knockdown of *Ire1* or *Atg7* expression had no effects upon the lifespan, climbing ability or viability of DA neurons (Fig. S9a–c). These results thus demonstrate IRE1 played a crucial role in promoting Parkinsonian neurodegeneration through autophagy-dependent loss of DA neurons.

## Discussion

PD is a devastating neurodegenerative disease with an age-related decline of motor functions, largely arising from selective loss of DA neurons in the substantia nigra pars compacta region^57,58^. As a pathological hallmark of PD, α-synuclein aggregation in Lewy bodies may reflect the ultimate consequence of the cellular machinery that goes awry for disposal of misfolded proteins. In this scenario, the overload of misfolded α-synuclein can lead to activation of the ER stress pathways, which is implicated in promoting cell death under various stress conditions^13^. However, the precise contribution of the individual UPR signaling branches during α-synucleinopathy in DA neurons remains unclear. Our results demonstrate in vivo that the IRE1 pathway is critical in coupling neuronal ER stress to autophagy-dependent neuron death, thereby driving the Parkinsonian neurodegeneration. These findings suggest that targeted inhibition of the IRE1 pathway or the resultant autophagy-dependent neuron death may provide a valuable intervention strategy against PD.

An important notion from our findings is that IRE1-induced autophagy-dependent neuron death may constitute a key component of the mechanism linking age-dependent accumulation of misfolded neurotoxic proteins to DA neuron loss and PD-like phenotypes. Autophagy is a cellular quality control mechanism for clearance of altered proteins and damaged organelles, and it is conceivable that dysfunction of autophagy in neurons can be associated with disruption of neuronal homeostasis^27^. Autophagy was also documented to be activated and exert cytoprotective effects during ER stress^29,59–61^. Surprisingly, our results clearly suggest that chronic activation of the IRE1 branch of the UPR can direct autophagy to the route of cell death in DA neurons in response to accumulation of α-synuclein. It is indicating that autophagy can serve as a “double-sword” mechanism by triggering cell death under certain pathological conditions, particularly when cellular protein degradation machinery fails to clear up misfolded protein wastes derived from imbalanced proteostasis. In addition, such IRE1-induced autophagy-dependent cell death may not be a neuron-specific phenomenon, and it has yet to be further deciphered if this occurs in a tissue- or cell-specific and context-dependent manner.

The IRE1-XBP1 branch of the UPR has long been thought to promote cell survival during ER stress, but the exact role of IRE1 versus XBP1 during neurodegeneration has yet to be clearly defined. Mouse model studies indicated that XBP1s was able to exert neuroprotective effects upon DA neurons in PD^21,44^, whereas XBP1 deficiency might protect against neurodegeneration in HD via its action upon FOXO1-regulated autophagy^23^. Our results in the *Drosophila* model of PD indicated an appreciable protection effect of XBP1 upon neuronal survival, which is in accordance with the reported findings in the mouse PD models^20^. However, we observed an XBP1-independent effect of IRE1 upon activation of neuronal autophagy in *Drosophila*. Based on the documented studies in mammalian cell lines^29^, IRE1 regulation of autophagy through the JNK pathway may operate as an evolutionarily conserved mechanism. Therefore, the regulatory actions of IRE1 versus XBP1 upon autophagy with regard to neuronal cell fate need to be dissected in the specific disease context.

In summary, our studies have uncovered a mechanism that the IRE1 pathway may act as a critical “rheostat” of proteostatic stress in the control of neuron cell fate in the context of α-synucleinopathy. IRE1 pathway-induced autophagy-dependent neuron death acts as a conserved pathogenic driver during PD progression, targeted modulation of IRE1 or autophgy in neurons may provide new avenues for developing therapeutics against this neurodegenerative disease.

## Methods

### Generation of transgenic flies

To generate transgenic UAS-IRE1and UAS-XBP1s flies, the cDNAs encoding *Drosophila* IRE1 andXBP1s were produced by RT-PCR using the total RNAs extracted from the w^1118^ line (#3605, from the Bloomington *Drosophila* Stock Center). The oligonucleotide primers used were as follows: IRE1, sense 5′-GGAAGATCTATGAGATTCTGCGTTGTTGTTTGTTGCG-3′, antisense 5′-CGGGGTACCTCACGTAGAATCGAGACCGAGGAGAGGGTTAGGGATAGGCTTACCTTCGAAATCCTGCGTTGAAGGTGGCAGCG-3′; The PCR products were subsequently subcloned into the pUAST plasmid for the expression of V5-tagged IRE1 protein. For Xbp1s, the PCR product was first subcloned into the PAC5.1 plasmid using the primers 5′-CGGGGTACCATGGCACCCACAGCAAACAC-3′, antisense 5′-CTAGTCTAGATCAGATCAAACTGGGAAACA-3′, and then subcloned into the pUAST plasmid using the primers 5′-CCGGAATCCATGGCACCCACAGCAAACAC-3′, antisense 5′-CGGGGTACCTCAATGGTGATGGTGATGATG-3′for the expression of V5-tagged XBP1s protein.

The pUAS-IRE1 or pUAS-XBP1s, vector was used to generate two independent UAS-IRE1 orUAS-XBP1s transgenic lines following standard germline transformation procedures^62^ at the Core Facility of Drosophila Resource and Technology, SIBCB, CAS. These lines were crossed with the special *Drosophila* line containing the double balancer (CyO/Bl; TM2, Ubx/TM6B, Tb) to identify the chromosome with the transgene insertion. The transgenic lines were backcrossed into the w^1118^ background for over five generations before further genetic manipulations.

### Fly strains and culture

The Gal4/UAS system^63^ was utilized for neuron-specific expression or RNAi knockdown of the desired genes. The GMR-Gal4 (stock number 8121) and Ddc-Gal4 driver lines were obtained from the Bloomington *Drosophila* Stock Center (BDSC; Department of Biology, Indiana University, Bloomington, IN). GMR-Gal4 or Ddc-Gal4 lines were crossed with w^1118^ flies to generate the GMR-Gal4 > + and Ddc-Gal4 > + control lines. The Dronc^I29^ line was kindly provided by Prof. Bertrand Mollereau at University of Lyon, France. The UAS-mCD8-GFP, UAS-GFP-mcherry-Atg8a, UAS-*Xbp1-*EGFP, UAS-puc^E69^, UAS-α-Syn, UAS-α-Syn^A30P^, UAS-α-Syn^A53T^, p35 and DRON^DN^ lines were as described previously^40,44,45,22,49,50,56^.

The transgenic RNAi lines were obtained from the Bloomington *Drosophila* Stock Center, including UAS-*Ire1*-Ri (stock number BS36743) and UAS-*Xbp1*-Ri (stock number BS25990), *Atg4-*Ri (stock number BS23542, BS28367), *Atg9-*Ri (stock number BS28057, BS34901), *Atg12-*Ri (stock number BS27752, BS34675), *Atg16-*Ri (stock number BS25652, BS34358), *Atg18-*Ri (stock number BS28061), *Fatp*-Ri (stock number BS50709, BS55919), and *Cds*-Ri (stock number BS28075, BS58118), or from the Vienna Drosophila RNAi Center (http://stockcenter.vdrc.at), including UAS-*Ire1*-Ri (stock number V39561), UAS-*Xbp1*-Ri (stock number V109312), and UAS-*Bsk*-Ri (stock number V34138, V34138). *Atg1-*Ri (V16133), *Atg5-*Ri(V104476), *Atg6-*Ri (stock number V25651), *Atg7-*Ri (stock number V27432, V45560), *Atg8a-*Ri (stock number V43096, V109654), *Atg10-*Ri (stock number V106317), *Atg13-*Ri (stock number V27956), *Atg17-*Ri (stock number V104864), *Atg101-*Ri (stock number V10617), and *Indy-*Ri (stock number V9981, V9982). Two RNAi lines for each target gene were used in the key experiments, and results from one line were presented.

All the fly lines were raised on standard yeast-cornmeal-agar food and maintained in vials at 25 °C with 50% humidity under a 12 h/12 h light/dark cycle.

### Immunoblotting, immunoprecipitation, and antibodies

Adult fly heads were extracted in RAPA buffer (150 mM NaCl, 1% NP-40, 0.5% sodium deoxycholate, 0.1% SDS, 50 mM Tris-HCl, pH 7.4) using a Tissuelyser-24 grinder (Jingxin, Shanghai, China). After centrifugation at 15,000*g* at 4 °C for 20 min, the supernatants were subjected to separation by SDS-PAGE before immunoblotting analysis. The following antibodies were used for immunohistochemistry, immunoprecipitation and immunoblotting: primary antibodies included mouse anti-V5 antibody (1:1000, Invitrogen, Catalogue no. 1461501), rabbit anti-phospho-IRE1α antibody (1:1000; Novus Biologicals, Catalogue no. NB100-2323), rabbit anti-phospho-JNK antibody (1:1000; Cell Signaling Technologies, Catalogue no.9251), anti-β-Gal antibody (1:500, Santa Cruze, Catalogue no. sc-65670), mouse anti-α-Tubulin antibody (1:10000; Sigma, Catalogue no. T6199), mouse anti-tyrosine hydroxylase (TH) antibody (1:1000, Immunostar, Catalogue no. 22941), Secondary antibodies included rabbit Alexa Fluor 568 or Alexa Fluor 488 goat anti-mouse IgG (Invitrogen).

Analysis of fly eyes and photoreceptor neurons. For analysis of external eyes by SEM, adult fly heads were dissected and directly examined by SEM. Images were taken at ×180 or ×800 magnification. For photoreceptor neuron analyses, fly heads were fixed in 4% formaldehyde for at least 24 h and embedded in Epon 812, followed by toluidine blue staining or TEM analysis. For toluidine blue staining, embedded fly eyes were semi-thin-sectioned at 500 μm and stained with toluidine blue as described^64^. For TEM analysis, the embedded eyes were ultrathin-sectioned at 70 nm followed by staining with Reynold’s lead citrate and 2% aqueous uranyl acetate. Retinal cells were subsequently analyzed and imaged using Tecnai G2 Spirit transmission electron microscope equipped with the GANTA-830 CCD camera.

### Immunohistochemistry

The eye imaginal discs from 3rd instar larvae were dissected in PBS and then fixed in 4% formaldehyde/PBS for 15 min before TUNEL (BrightRed Apoptosis Detection Kit, Vazyme) or immunofluorescence staining. For immunostaining, mouse anti-V5 antibody (1:1000; Invitrogen, Catalogue no. 1461501) or rabbit antiRef(2)P antibody (1:200; Abcam, Catalogue no. ab178440) was used as the primary antibody, and rabbit Alexa Fluor 568 or Alexa Fluor 488 goat anti-mouse IgG (Invitrogen) as the secondary antibody. Tissues were mounted in the anti-fading mounting medium with DAPI (Prolong Antifade; Invitrogen). Fluorescent images were obtained by confocal laser-scanning microscopy (Olympus BX61).

### Lifespan and locomotor activity

Cohorts of 90 flies for each genotype were monitored for survival. Mortality was scored every three days. Climbing ability of flies was measured as described^65^. Briefly, 90 flies of both sexes for each genotype were tapped to the bottom of a graduated cylinder (1.5 cm in diameter; 25 cm in length). Flies that could climb up to or above 5-cm from the bottom of the cylinder within 10 s were counted.

### Quantification of DA neurons

Fly brains were dissected and subjected to whole-mount immunostaining using the anti-TH antibody as described previously^65^. Brains were examined by confocal laser-scanning microscope (Olympus BX61), and the numbers of TH-positive neurons in all DA clusters within a half of the brain were counted, except those in the paired anterolateral medial (PAM) cluster in which the high fluorescent intensity did not allow for precise counting due to the high density of DA neurons.

### Quantitative RT-PCR

Total RNAs were prepared from adult heads using the TRIzol reagent (Invitrogen). cDNAs were synthesized with M-MLV reverse transcriptase and random hexamer primers (Invitrogen). SYBRGreen was purchased from Biotool (Houston, TX, USA). Real-time quantitative PCR was conducted using the 7500 FAST Real-Time PCR System (Applied Biosystems). For normalization, *rp49* was utilized as the internal control. The oligonucleotide primers used were as follows:

*rp49*: sense 5′-TCCTACCAGCTTCAAGATGACC-3′, antisense 5′-CACGTTGTGCACCAGGAACT-3′;

*Bip*: sense 5′-GATTTGGGCACCACGTATTCC-3′, antisense 5′-GGAGTGATGCGGTTACCCTG-3′;

*Edem1*: sense 5′-ACGCCTACGATGGTTACCTG-3′, antisense 5′-ACACGTTGATGTCCCTGTCA-3′; *Edem2*: sense 5′-CTTAGCACCGAAACCACCAT-3′, antisense 5′-ACTCCTCGGTACCGTCCTTT-3′;

*Xbp1s*: sense 5′-ACCAACCTTGGATCTGCCG-3′, antisense 5′-CGCCAAGCATGTCTTGTAGA-3′;

*Total Xbp1*: sense 5′-TGGGAGGAGAAAGTGCAAAG-3′, antisense 5′-TCCGTTCTGTCTGTCAGCTC-3′;

*Atg1*: sense 5′-GAGTATTGCAATGGCGGCGACT-3′, antisense 5′-CAGGAATCGCGCAAACCCAA-3′;

*Atg3*: sense 5′- TCTTCCAGGTCCCAATATGGCC-3′, antisense 5′-TGAAAAGCATGGCGGGTCTT-3′;

*Atg5*: sense 5′- GCACGCACGGCATTGATCTACA-3′,antisense 5′-GCCCTGGGATTTGCTGGAAT-3′;

*Atg6*: sense 5′-TATGTTGAGGTGCTCGGCGAGA-3′, antisense 5′-TGGTCCACTGCTCCTCCGAGTT-3′;

*Atg7*: sense 5′- TTTTGCCTCACTCCATCCGTGG-3′, antisense 5′-ATCCTCGTCGCTATCGGACATG-3′;

*Atg8b*: sense 5′- TTCGAACCGTATTCCAGTCCGC-3′, antisense 5′-TCGTCGGGACGCAGATTGAT-3′;

*Atg12*: sense 5′-GCAGAGACACCAGAATCCCAG-3′, antisense 5′-GTGGCGTTCAGAAGGATACAAA-3′;

*Atg18a*: sense 5′-GGTGATGGCAAGTCGGCTGTTT-3′, antisense 5′-ATCATCATTATCGCCGCCGC-3′;

*Fatp*: sense 5′- CCTTGGAAGTCTGGTAGTACAGCG-3′, antisense 5′- AGAATGTTTCCACCAGCGAGGTGG-3′;

*Cds*: sense 5′-GAAGTTCCTGGTGACCTATCACCG-3′, antisense 5′-GTCTTCTTGGGGCTGAGCTTGATG-3′;

*Indy*: sense 5′- GCTTGCGGGTGAAGTACATCACAACC -3′, antisense

5′- TACCTTCAAGGGCATCTACGAGGC -3′;

CG3984: sense 5′-CTACTGTTGTTCCTGGTACCCC-3′, antisense

5′- CTGGTTGCTCAGTAACACTTGG -3′;

CG5888: sense 5′ ACTCGGTTGGCACATTATCACCGC -3′, antisense

5′- GGAAGCAGTGATTTGCCCGGTAAC -3′;

CG6650: sense 5′- ACAATGGGACAGGCAAAGAC -3′′, antisense

5′- GGTGACATTCGTTTCCGAGT-3′;

*Hydr2*: sense 5′-CGCATACACGACTATTTAACGC -3′, antisense

5′- TTTGGTTTCTCTTTGATTTCCG -3′;

*Crc*: sense 5′ - TGCGAGTCTCAGGCGTCTTCATCTT-3′, antisense 5′- CTGCTCGATGGTTGTAGGAGCTGG -3′;

*Atf6*: sense 5′ - AACGTAATTCCACGGAAGCCCAACA-3′, antisense 5′- GCGACGGTAGCTTGATTTCTAGAGCC -3′;

*PEK*: sense 5′- TCTGGTCATTGAACGTCATGTGCCTG-3′, antisense 5′-TGATTTGCTTGTCCAGGTGGGAAGC-3′.

### Statistical analysis

All data are presented as the mean ± standard errors of the mean (s.e.m) from at least three independent experiments. Statistical analysis was performed using unpaired two-tailed Student’s *t* test, one-way or two-way analysis of variance (ANOVA) followed by Bonferroni’s post test with GraphPad Prism 5.0. *P* < 0.05 was considered to be statistically significant.

## Supplementary information

Supplemental Figure 1

Supplemental Figure 2

Supplemental Figure 3

Supplemental Figure 4

Supplemental Figure 5

Supplemental Figure 6

Supplemental Figure 7

Supplemental Figure 8

Supplemental Figure 9

supplementary figure legends
