# Correction: IRE1 promotes neurodegeneration through autophagy-dependent neuron death in the *Drosophila* model of Parkinson’s disease

**DOI:** 10.1038/s41419-020-2346-y

**Published:** 2020-02-24

**Authors:** Cheng Yan, Jingqi Liu, Jiamei Gao, Ying Sun, Lei Zhang, Haiyun Song, Lei Xue, Lixing Zhan, Guanjun Gao, Zunji Ke, Yong Liu, Jingnan Liu

**Affiliations:** 1 Key Laboratory of Nutrition and Metabolism, Institute for Nutritional Sciences, Shanghai Institutes for Biological Sciences, Chinese Academy of Sciences, Shanghai, 200031 China; 2 School of Life Science and Technology, ShanghaiTech University, Shanghai, 201210 China; 3 The State Key Laboratory of Cell Biology, CAS Center for Excellence in Molecular Cell Science, Innovation Center for Cell Signaling Network, Institute of Biochemistry and Cell Biology, Shanghai Institutes for Biological Sciences, Chinese Academy of Sciences, Shanghai, 200031 China; 4 School of Life Science and Technology, Tongji University, Shanghai, 200092 China; 5 Department of Biochemistry, Basic Medical College, Shanghai University of Chinese Traditional Medicine, Shanghai, 201203 China; 6 Hubei Key Laboratory of Cell Homeostasis, College of Life Sciences; the Institute for Advanced Studies, Wuhan University, Wuhan, 430072 China; 7 Present Address: School of Medicine, Xinxiang University, Xinxiang, Henan 453003 China

**Keywords:** Macroautophagy, Cell death in the nervous system, Parkinson's disease

**Correction to: Cell Death & Disease**

10.1038/s41419-019-2039-6 published online 22 October 2019

Since online publication of this article, the authors noticed that some of the fly stocks were mislabelled in the methods and in Supplementary Figure 5. The corrected methods text is provided below:

The transgenic RNAi lines were obtained from the Bloomington Drosophila Stock Center, including UAS-Ire1-Ri (stock number BS36743), UAS-Xbp1-Ri (stock number BS25990), Atg4aMB03551 and Atg4a-Ri (stock number BS23542, BS28367), Atg6-Ri (BS28060), Atg8b-Ri(stock number BS27554), Atg9-Ri (stock number BS28055, BS34901), Atg12-Ri (stock number BS27552, BS34675), Atg16-Ri (BS34358), Atg18-Ri (stock number BS28061), Fatp-Ri (stock number BS50709, BS55919), and Cds-Ri (stock number BS28075, BS58118), or from the Vienna Drosophila RNAi Center (http://stockcenter.vdrc.at), including UAS-Ire1-Ri (stock number V39561), UAS-Xbp1-Ri (stock number V109312), UAS-Bsk-Ri (stock number V34138, V34139), Atg1-Ri (V16133), Atg5-Ri(V104461), Atg16-Ri (stock number V25652), Atg7-Ri (stock number V27432, V45560), Atg8a-Ri (stock number V43096, V109654), Atg8b-Ri (stock number V17079), Atg10-Ri (stock number V106317), Atg13-Ri (stock number V27956), Atg17-Ri (stock number V104864), Atg101-Ri (stock number V106176), and Indy-Ri (stock number V9981, V9982). Two RNAi lines for each target gene were used in the key experiments, and results from one line were presented.

**Figure Supplemental 5.**
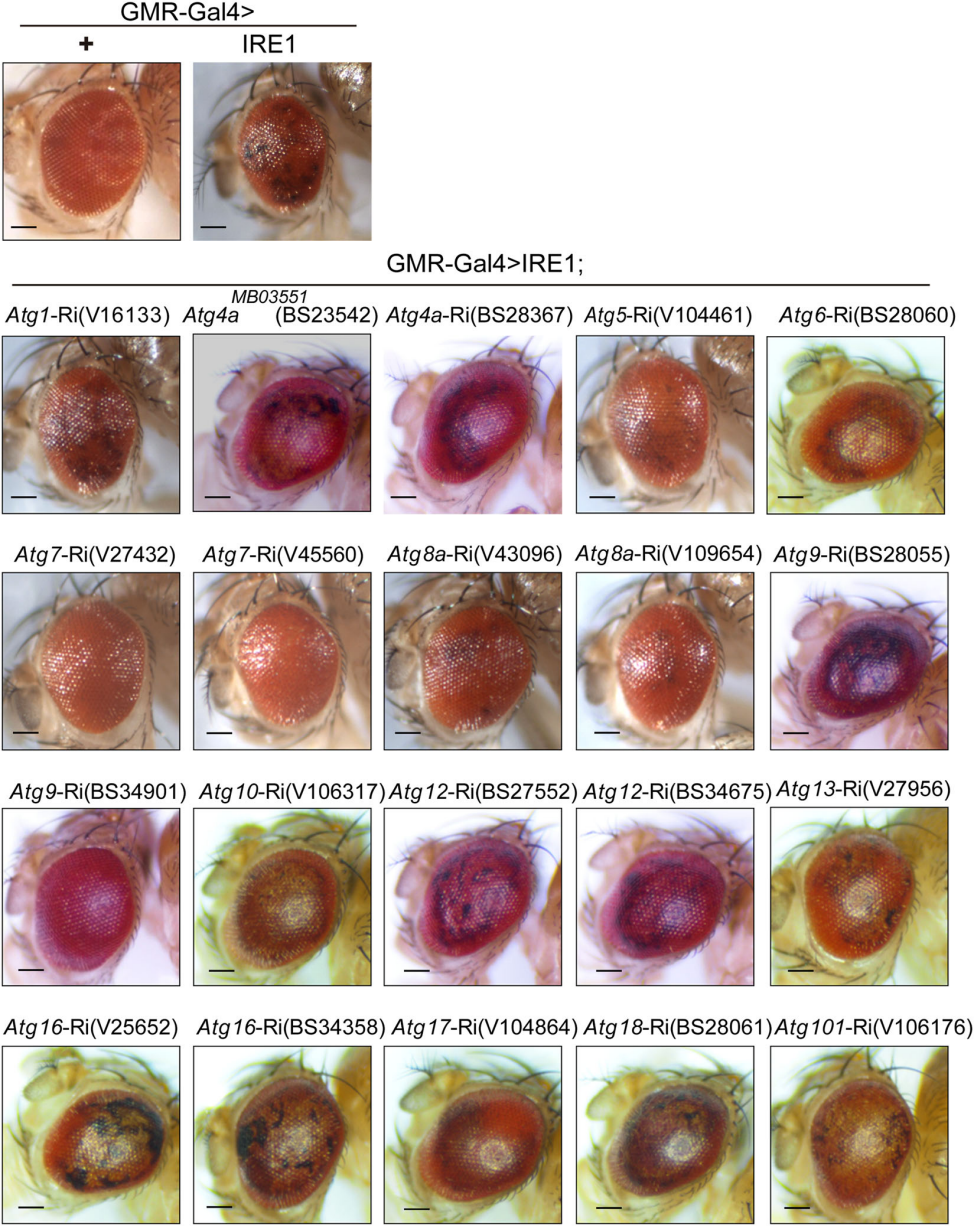
Effects of knockdown of Atg family gene expression on IRE1-induced neuron death. Representative light microscopy images of external eyes from adult GMR-Gal4>+ and GMR-Gal4>IRE1 flies versus GMRGal4> IRE1;*Atg1*-Ri(V16133), GMR-Gal4>IRE1;*Atg4a*MB03551 (BS23542), GMR-Gal4>IRE1;*Atg4a*-Ri(BS28367), GMR-Gal4>IRE1;*Atg5*-Ri(V104461), GMR-Gal4>IRE1;*Atg6*-Ri(BS28060), GMR-Gal4>IRE1;*Atg7*-Ri(V27432), GMR-Gal4>IRE1;*Atg7*-Ri(V45560), GMR-Gal4>IRE1;*Atg8a*-Ri(V43096), GMR-Gal4>IRE1;*Atg8a*-Ri(V109654), GMR-Gal4>IRE1;*Atg9*-Ri(BS28055), GMR-Gal4>IRE1;*Atg9*-Ri(BS34901), GMR-Gal4>IRE1;*Atg10*-Ri(V106317), GMR-Gal4>IRE1;*Atg12*-Ri(BS27552), GMR-Gal4>IRE1;*Atg12*-Ri(BS34675), GMR-Gal4>IRE1;*Atg13*-Ri(V27956), GMR-Gal4>IRE1;*Atg16*-Ri(V25652), GMR-Gal4>IRE1;*Atg16*-Ri(BS34358), GMR-Gal4>IRE1;*Atg17*-Ri(V104864), GMR-Gal4>IRE1;*Atg18*-Ri(BS28061), and GMR-Gal4>IRE1;*Atg101*-Ri(V106176) flies (*n* = 5–8 flies/genotype). Scale bar represents 100 μm.

In addition, Supplementary Figure 5 contained incorrectly labelled fly stocks. The corrected figure and legend have been provided below:

The authors apologise for this error.

The incorrect supplementary figure has now been replaced.

