# Supplementary figures and images for: IRE1 promotes neurodegeneration through autophagy-dependent neuron death in the Drosophila model of Parkinson’s disease
Source: Cell Death Dis. 2019 Oct 22;10(11):800. doi: 10.1038/s41419-019-2039-6 (PMC6805898; doi:10.1038/s41419-019-2039-6)

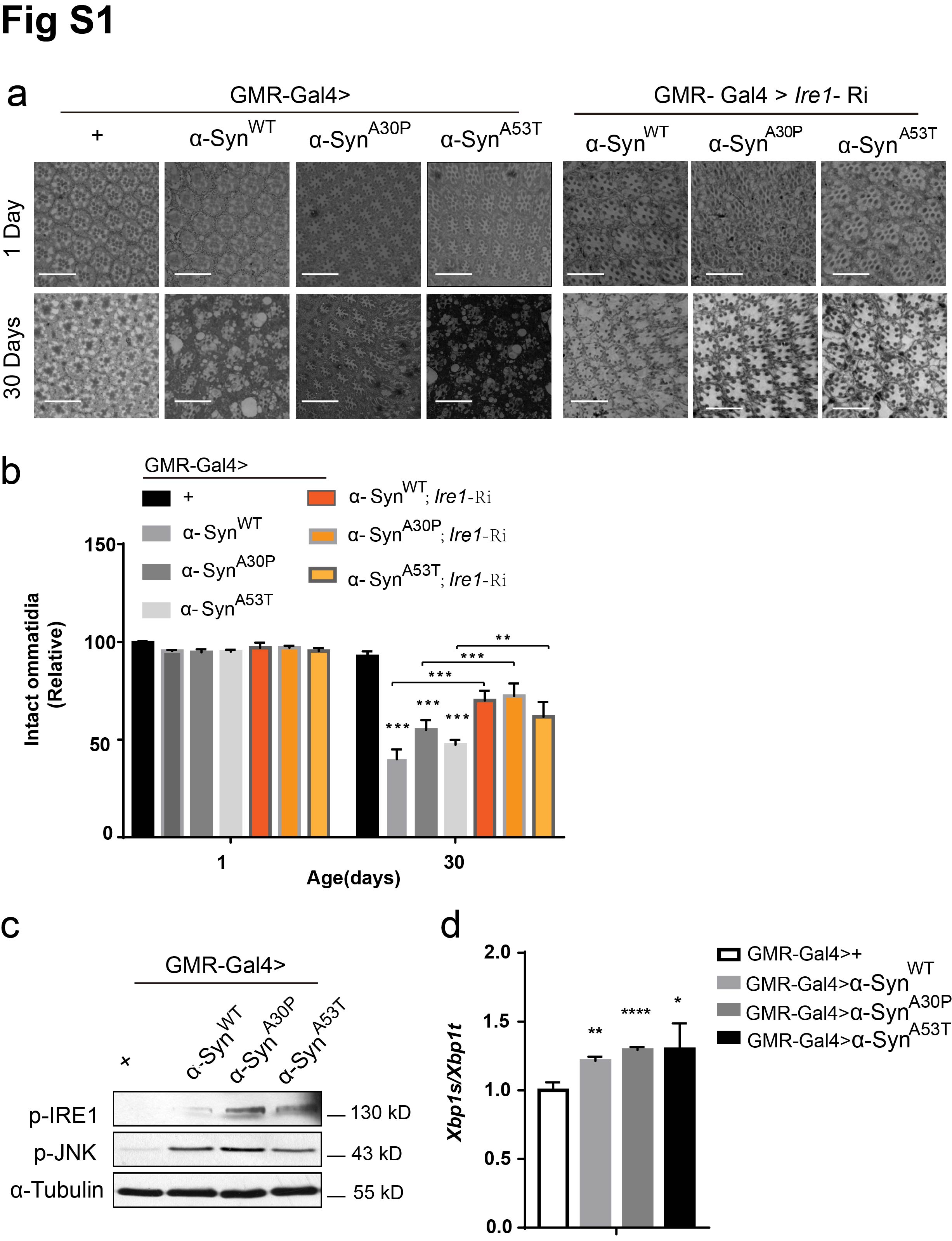

Supplement: Supplementary file 1 — Supplemental Figure 1 [file 41419_2019_2039_MOESM1_ESM.jpg]

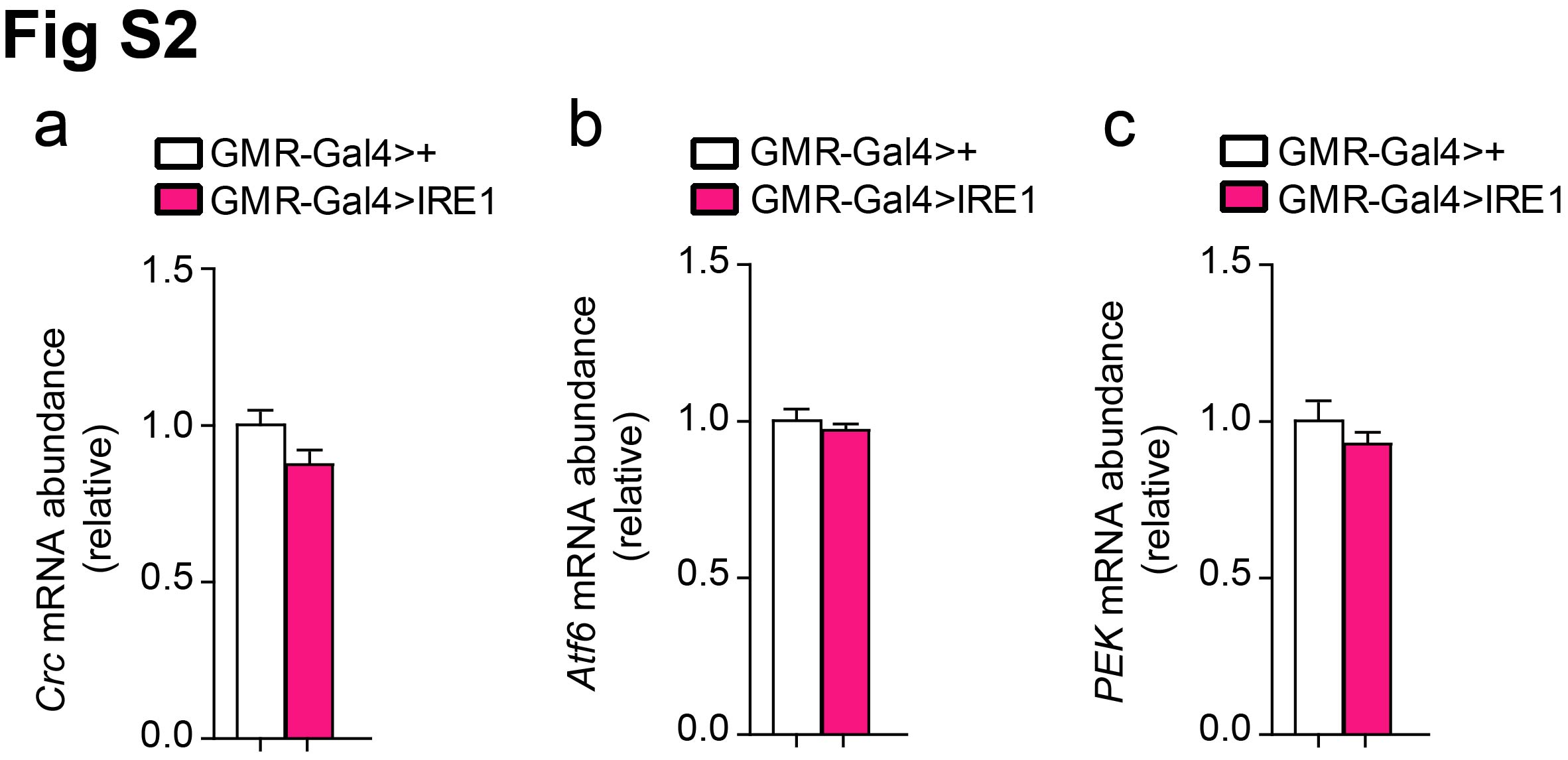

Supplement: Supplementary file 2 — Supplemental Figure 2 [file 41419_2019_2039_MOESM2_ESM.jpg]

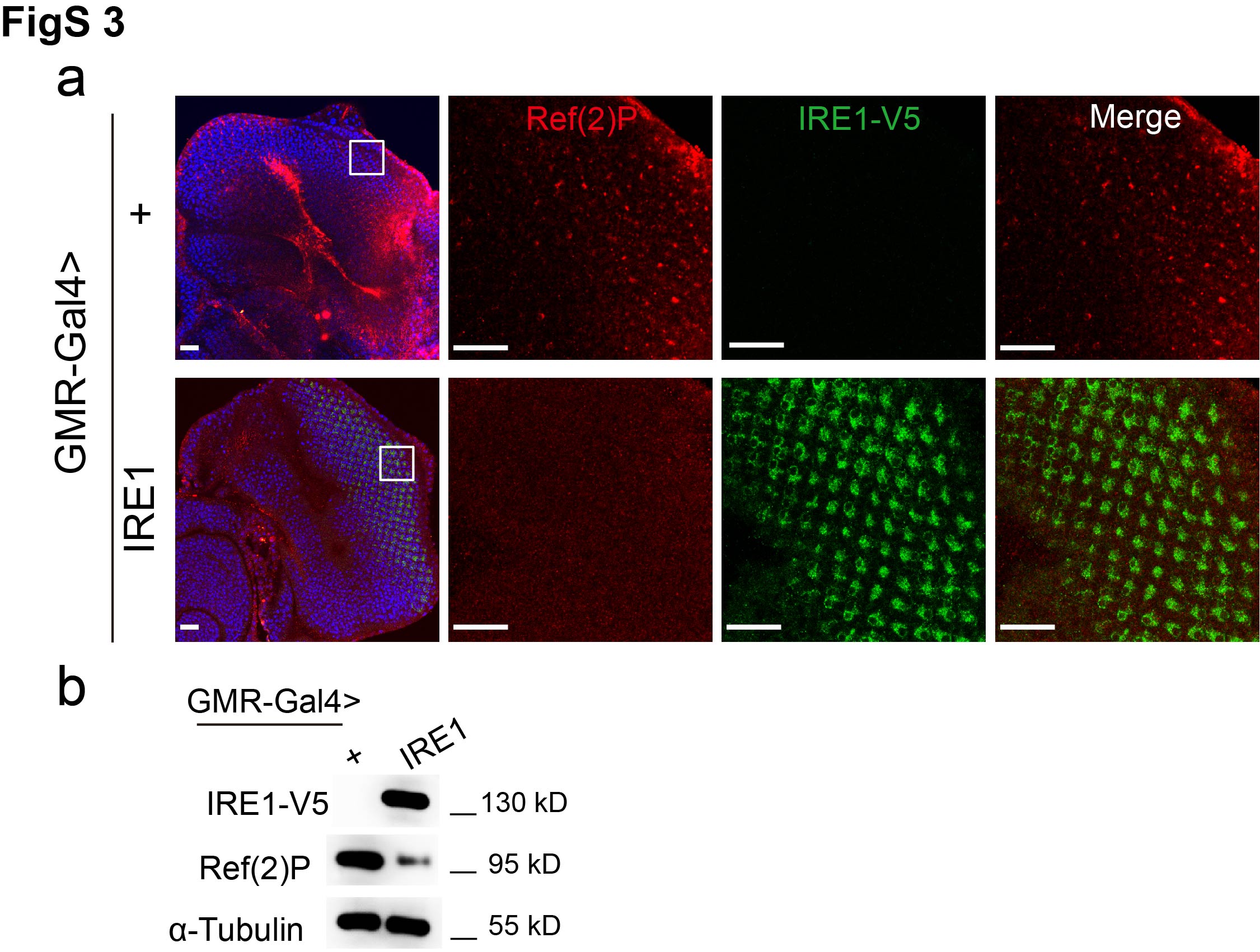

Supplement: Supplementary file 3 — Supplemental Figure 3 [file 41419_2019_2039_MOESM3_ESM.jpg]

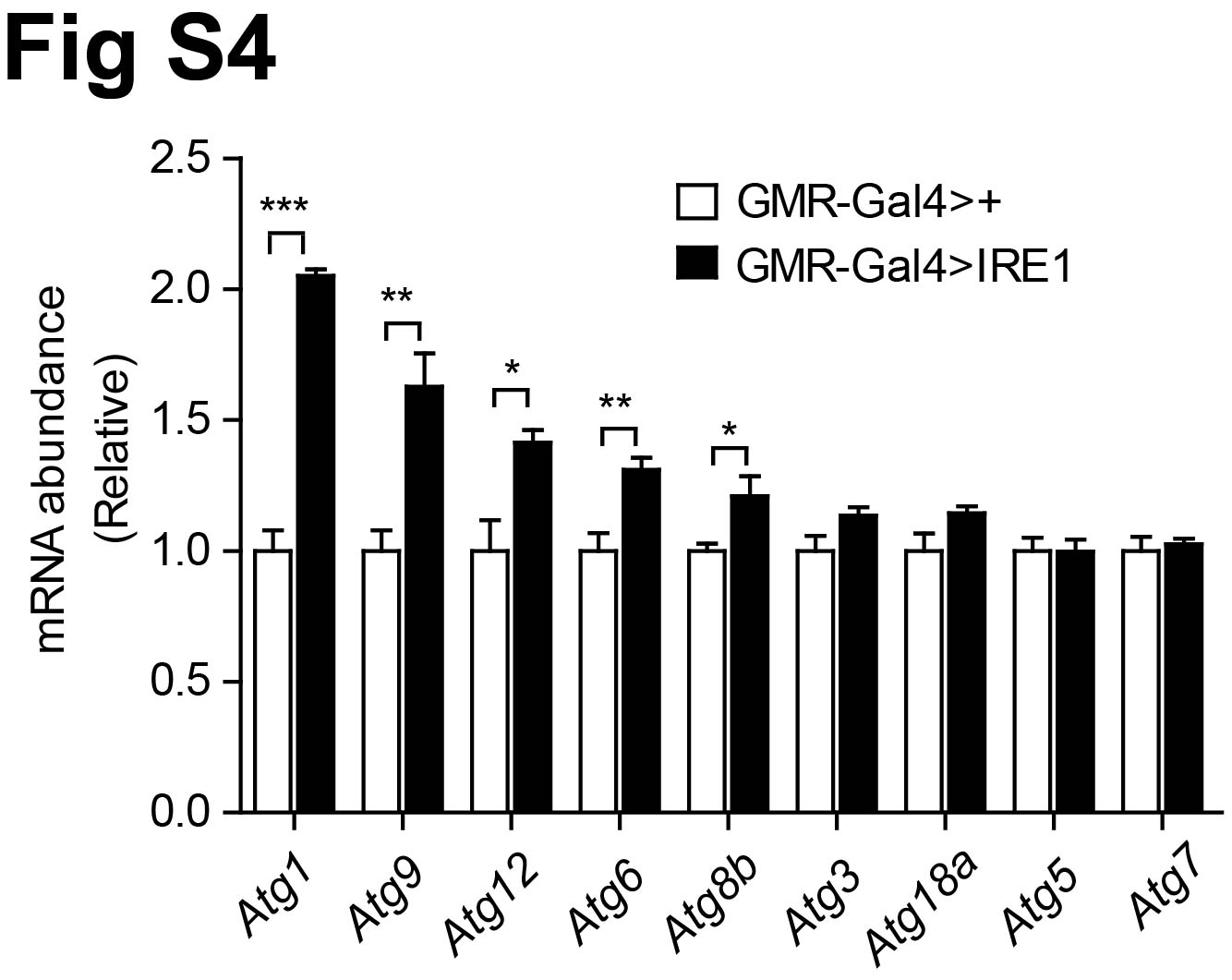

Supplement: Supplementary file 4 — Supplemental Figure 4 [file 41419_2019_2039_MOESM4_ESM.jpg]

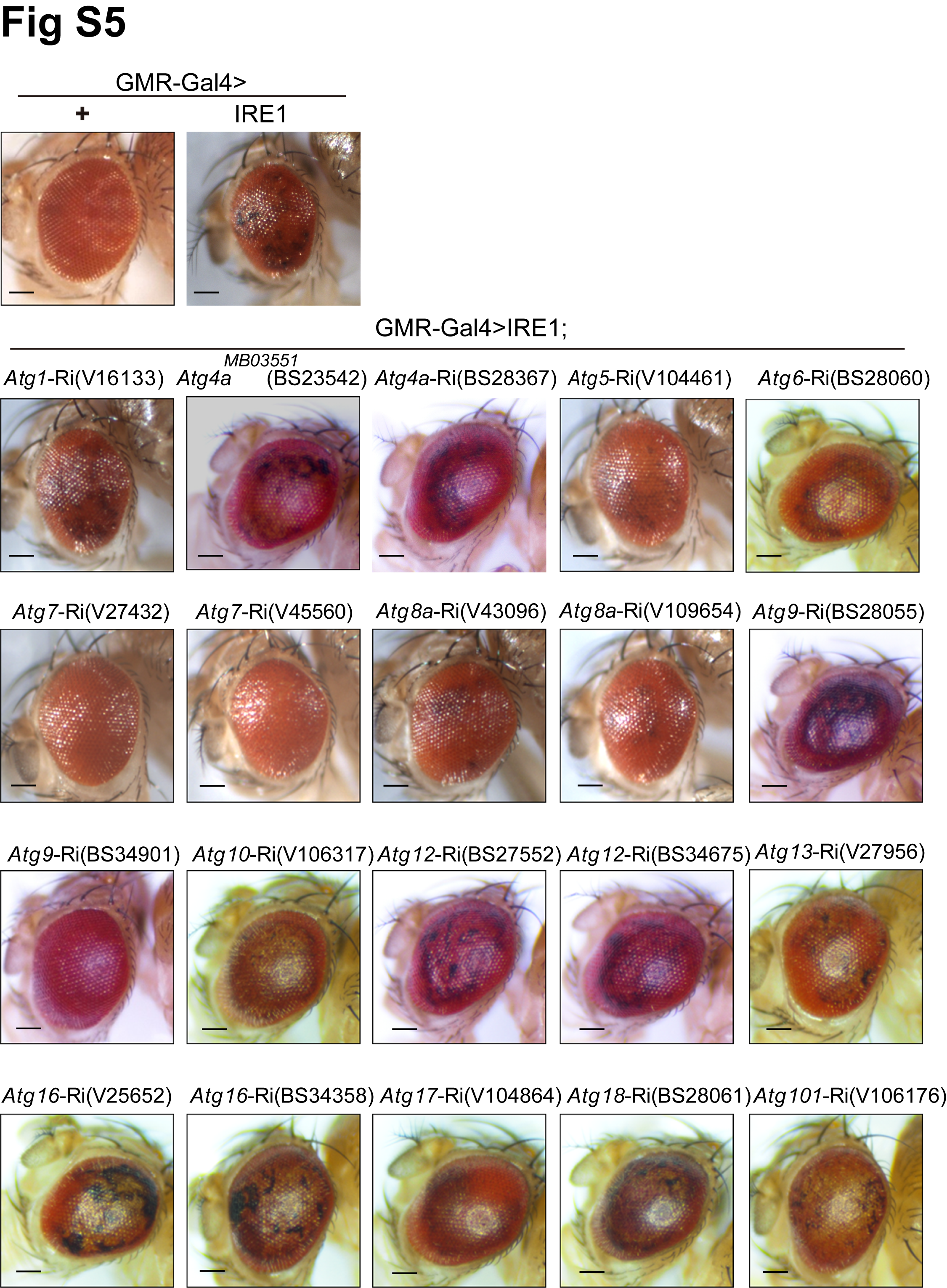

Supplement: Supplementary file 5 — Supplemental Figure 5 [file 41419_2019_2039_MOESM5_ESM.tif]

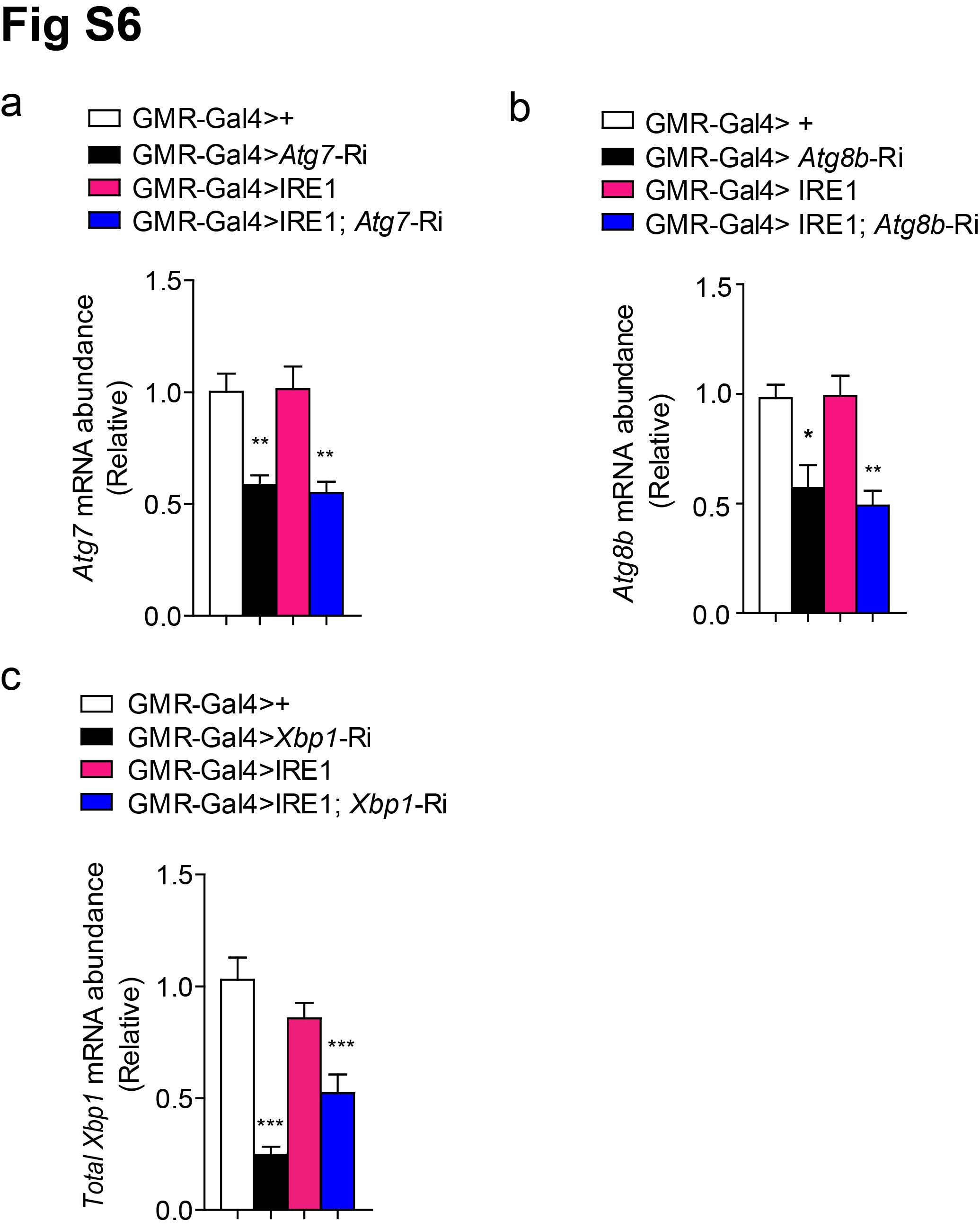

Supplement: Supplementary file 6 — Supplemental Figure 6 [file 41419_2019_2039_MOESM6_ESM.jpg]

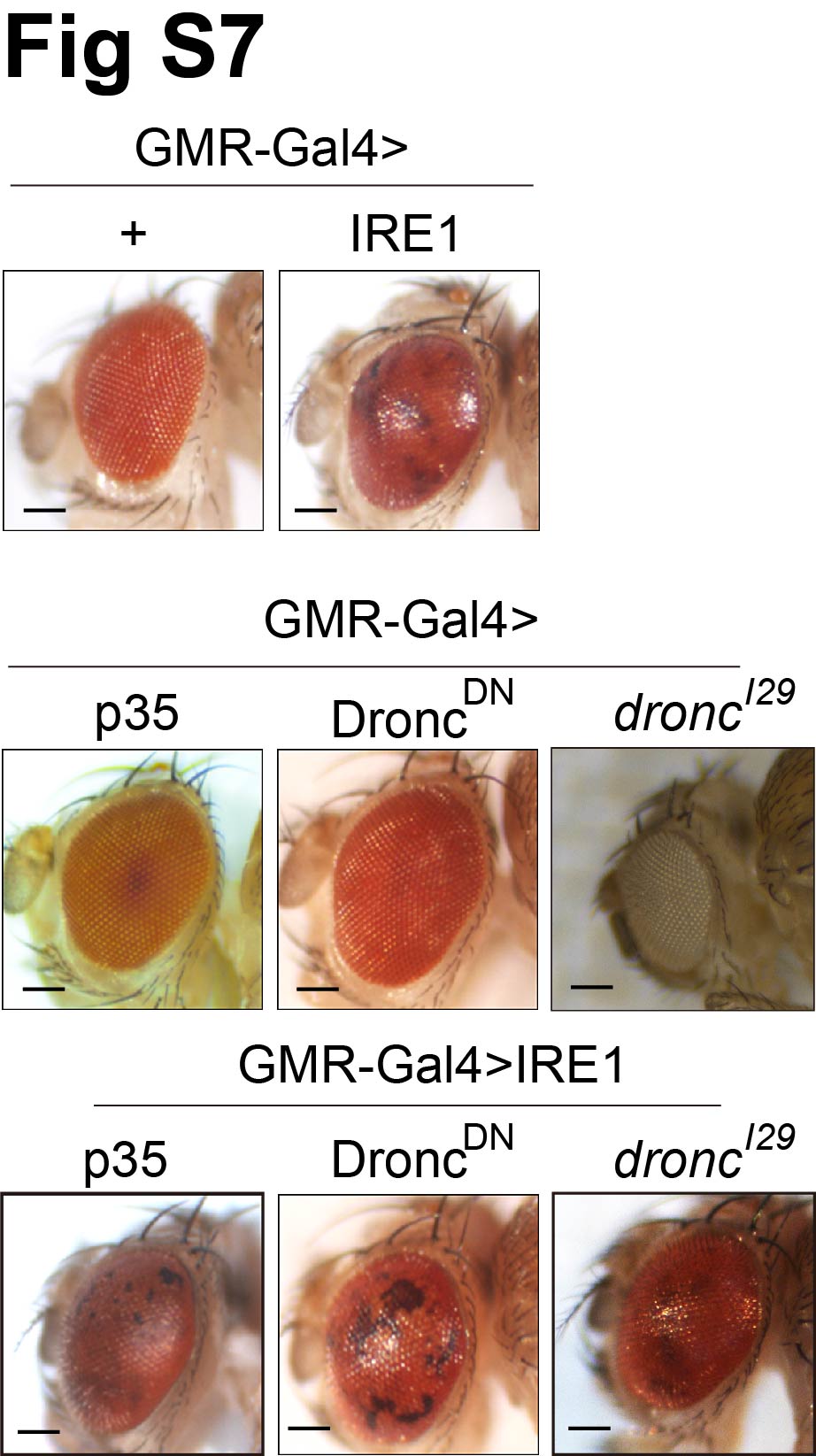

Supplement: Supplementary file 7 — Supplemental Figure 7 [file 41419_2019_2039_MOESM7_ESM.jpg]

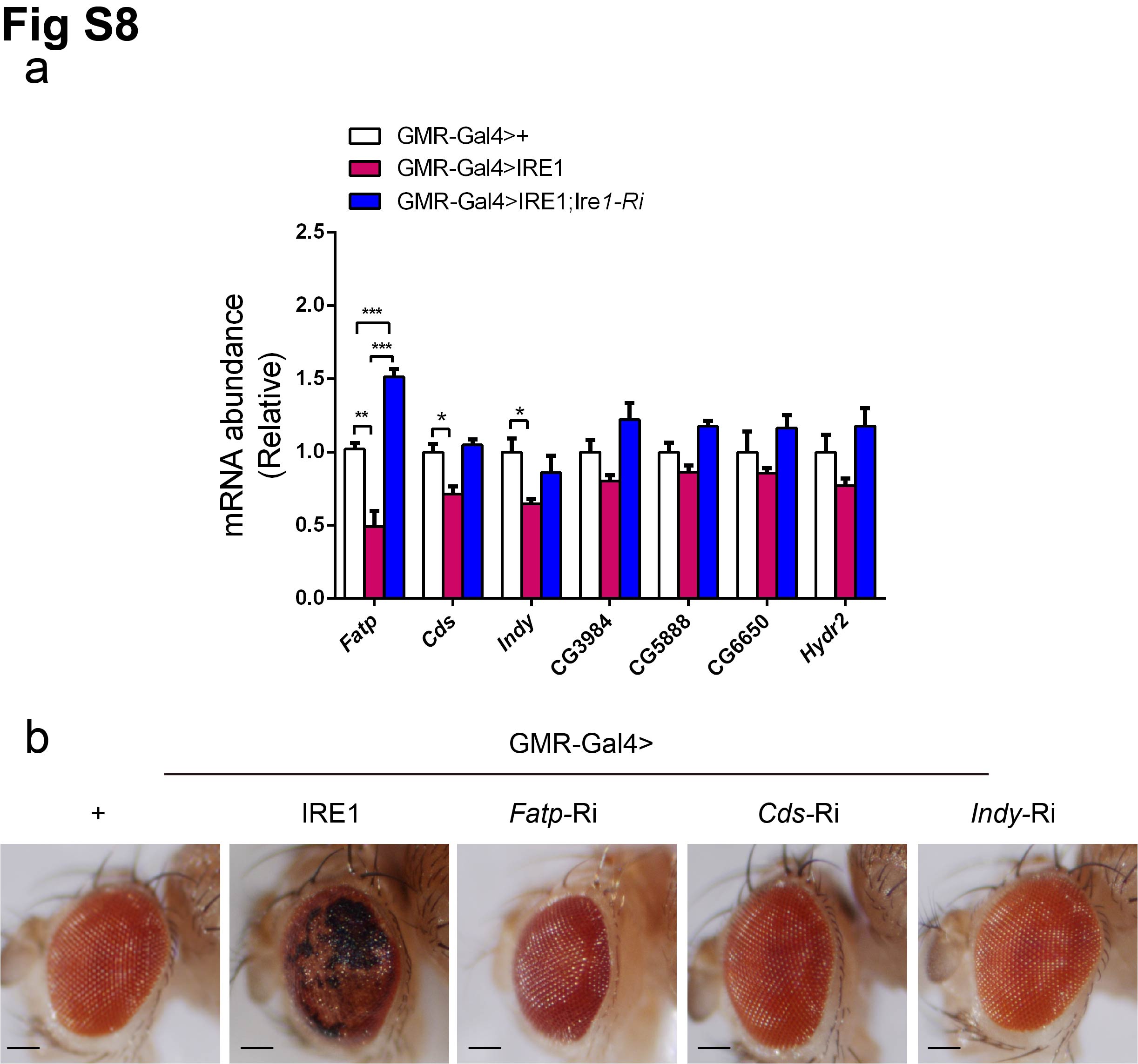

Supplement: Supplementary file 8 — Supplemental Figure 8 [file 41419_2019_2039_MOESM8_ESM.jpg]

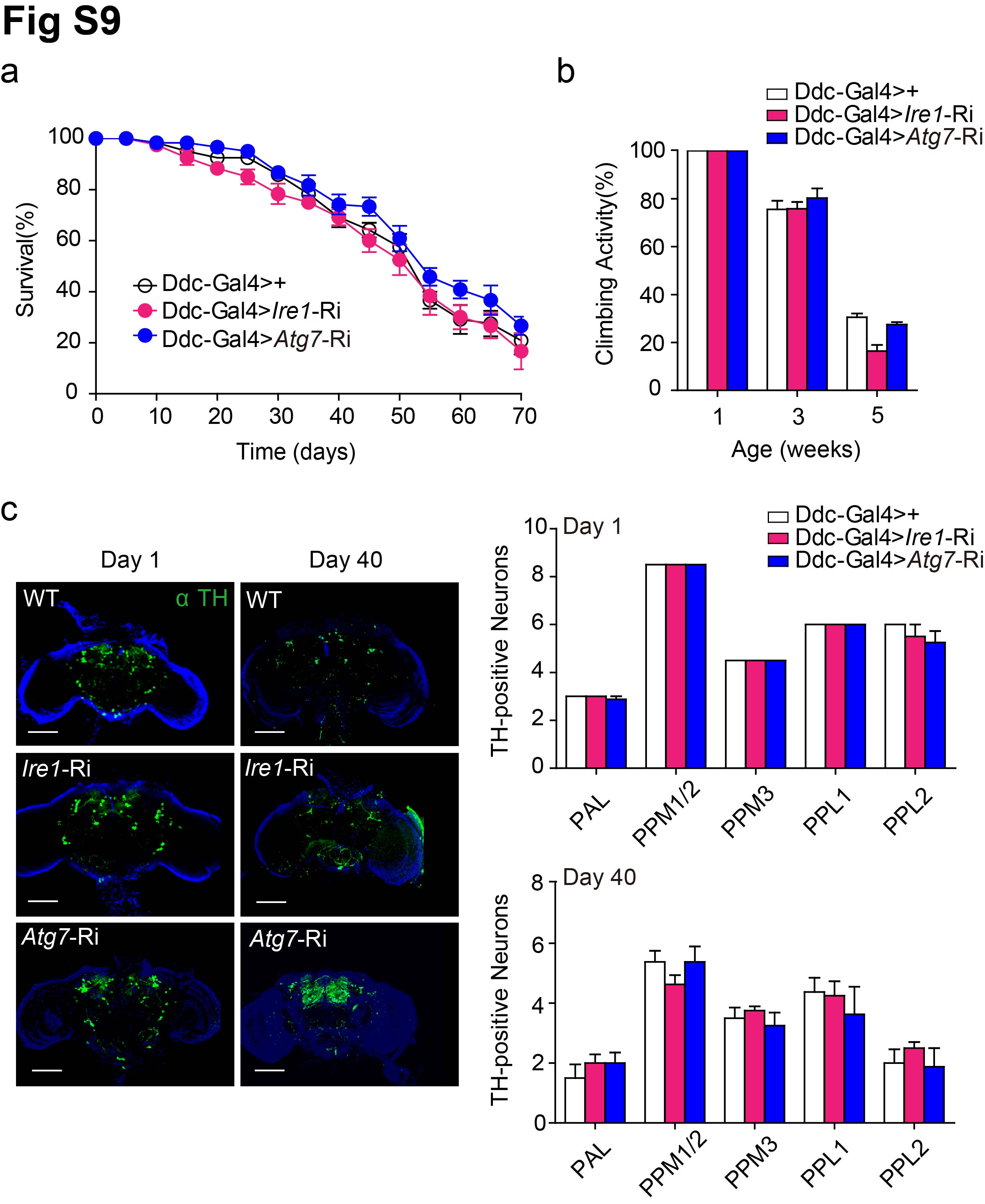

Supplement: Supplementary file 9 — Supplemental Figure 9 [file 41419_2019_2039_MOESM9_ESM.jpg]
