# Supplementary material for: IRE1 promotes neurodegeneration through autophagy-dependent neuron death in the Drosophila model of Parkinson’s disease
Source: Cell Death Dis. 2019 Oct 22;10(11):800. doi: 10.1038/s41419-019-2039-6 (PMC6805898; doi:10.1038/s41419-019-2039-6)
Supplement: Supplementary file 10 — supplementary figure legends [file 41419_2019_2039_MOESM10_ESM.doc]

**IRE1 Promotes Neurodegeneration through Autophagy-dependent Neuron Death in the *Drosophila* Model of Parkinson’s Disease**

Cheng Yan^1,7,8^, Jingqi Liu^1,8^, Jiamei Gao^2,8^, Ying Sun^1^, Lei Zhang^2,3^, Haiyun Song^1^, Lei Xue^4^, Lixing Zhan^1^, Guanjun Gao^2^, Zunji Ke^5^, Yong Liu^*,6^, and Jingnan Liu^*,1,2^

**Supplementary figure legends**

**Supplemental Figure 1. IRE1 is associated with α-Synuclein-induced neurodegeneration.** (**a**) Representative light microscopy images of tangential sections of adult eyes stained with toluidine blue. Eyes were analyzed for the GMR-Gal4>+ control versus GMR-Gal4>α-Syn^WT^, GMR-Gal4>α-Syn^A30P^, GMR-Gal4>α-Syn^A53T^, GMR-Gal4>*Ire1*-Ri;α-Syn^WT^, GMR-Gal4>*Ire1*-Ri;α-Syn^A30P^, and GMR-Gal4> *Ire1*-Ri;α-Syn^A53T^ lines at 1 or 30 days after eclosing from the pupal case (n=3-5 flies/genotype). Scale bar represents 20 µm. (**b**) Quantification of photoreceptor loss in tangential sections shown as the percentage of intact ommatidia with a preserved complement of 7 rhabdomeres at two time points, respectively. Data are presented as mean ± s.e.m. (n=3-5 flies/genotype). ***P<*0.01, ****P<*0.001 by two-way ANOVA. (**c**) Immunoblot analysis of the phosphorylation of IRE1 and JNK from the adult head lysates of the indicated genotypes at 30 days of age (n=30 flies/genotype; representative of two independent experiments). α-Tubulin was used as the internal control. (**d**) Quantitative RT-PCR analysis of *Xbp1* mRNA splicing from the head lysates of adult GMR-Gal4>α-Syn^WT^, GMR-Gal4>α-Syn^A30P^, GMR-Gal4>α-Syn^A53T^ or GMR-Gal4>+ control flies. Data are shown as mean ± s.e.m. (n=30 flies/genotype; two independent experiments). **P<*0.05, ***P<*0.01, *****P<*0.0001 by Student’s *t*-test.

**Supplemental Figure 2. Quantitative RT-PCR analysis of the expression of UPR signaling genes.** (**a-c**) The abundance of adult head *Crc* (**a**), *Atf6* (**b**), and *PEK* (**c**) mRNA was determined for GMR-Gal4>+ and GMR-Gal4>IRE1 flies. All data are shown as mean ± s.e.m. (n=30 flies/genotype; at least two independent experiments).

**Supplemental Figure 3. IRE1 enhances autophagic flux in fly photoreceptor neuron. (a)** Analysis of autophagy flux by immunostaining of Ref(2)p in eye discs from 3^rd^ instar larvae of the GMR-Gal4>+ and GMR-Gal4>IRE1 flies. Representative confocal images are shown, along with IRE1 immunostaining using anti-V5 antibody and DAPI staining with the enlarged regions indicated (n=10 flies/genotype). Scale bar represents 30 µm. (**b**) Immunoblot analysis of Ref(2)p from the adult head lysates of the indicated genotypes at 14 days of age (n=30 flies/genotype; representative of two independent experiments). α-Tubulin was used as the internal control.

**Supplemental Figure 4. Quantitative RT-PCR analysis of the expression of *Atg* family genes.** The abundance of adult head mRNAs encoding the indicated *Atg* genes was determined for GMR-Gal4>+ and GMR-Gal4>IRE1 flies. All data are shown as mean ± s.e.m. (n=30 flies/genotype; at least three independent experiments). **P<*0.05, ***P<*0.01, ****P<*0.001 by Student’s *t*-test.

**Supplemental Figure 5. Effects of knockdown of *Atg* family gene expression on IRE1-induced neuron death.** Representative light microscopy images of external eyes from adult GMR-Gal4>+ and GMR-Gal4>IRE1 flies versus GMR-Gal4>IRE1;*Atg1-*Ri(V16133), GMR-Gal4>IRE1;*Atg4-*Ri(BS23542), GMR-Gal4>IRE1;*Atg4-*Ri(BS28367), GMR-Gal4>IRE1;*Atg5-*Ri(V104476), GMR-Gal4>IRE1;*Atg6-*Ri(V25651), GMR-Gal4>IRE1;*Atg7-*Ri(V27432), GMR-Gal4>IRE1;*Atg7-*Ri(V45560), GMR-Gal4>IRE1;*Atg8a-*Ri(V43096), GMR-Gal4>IRE1;*Atg8a-*Ri(V109654), GMR-Gal4>IRE1;*Atg9-*Ri(BS28057), GMR-Gal4>IRE1;*Atg9-*Ri(BS34901), GMR-Gal4>IRE1;*Atg10-*Ri(V106317), GMR-Gal4>IRE1;*Atg12-*Ri(BS27752), GMR-Gal4>IRE1;*Atg12-*Ri(BS34675), GMR-Gal4>IRE1;*Atg13-*Ri(V27956), GMR-Gal4>IRE1;*Atg16-*Ri(BS25652), GMR-Gal4>IRE1;*Atg16-*Ri(BS34358), GMR-Gal4>IRE1;*Atg17-*Ri(V104864), GMR-Gal4>IRE1;*Atg18-*Ri(BS28061), and GMR-Gal4>IRE1;*Atg101-*Ri(V10617) flies (n=5-8 flies/genotype). Scale bar represents 100 µm.

**Supplemental Figure 6. Quantitative RT-PCR analysis of the expression of genes targeted for RNAi knockdown.** (**a**) The abundance of adult head *Atg7* mRNA was determined for GMR-Gal4>+, GMR-Gal4>*Atg7*-Ri, GMR-Gal4>IRE1 and GMR-Gal4>IRE1;*Atg7*-Ri flies. (**b**) The abundance of adult head *Atg8b* mRNA was determined for GMR-Gal4>+, GMR-Gal4>*Atg7*-Ri, GMR-Gal4>IRE1 and GMR-Gal4>IRE1;*Atg7*-Ri flies. (**c**) The abundance of adult head *Xbp1* mRNA was determined for GMR-Gal4>+, GMR-Gal4>*Xbp1*-Ri, GMR-Gal4>IRE1 and GMR-Gal4>IRE1;*Xbp1*-Ri flies. All data are shown as mean ± s.e.m. (n=30 flies/genotype; at least three independent experiments). **P<*0.05, ***P<*0.01, ****P<*0.001 by one-way ANOVA.

**Supplemental Figure 7. Blocking apoptosis in photoreceptor neurons does not affect IRE1-induced neuron death.** Representative light microscopy images of external eyes from adult GMR-Gal4>IRE1, GMR-Gal4>IRE1;p35, GMR-Gal4>IRE1;Dronc^DN^ flies, GMR-Gal4>IRE1;*dronc^I29^* versus GMR-Gal4>+, GMR-Gal4>p35, GMR-Gal4>Dronc^DN^, and GMR-Gal4> *dronc^I29^* flies (n=5-8 flies/genotype). Scale bar represents 100 µm.

**Supplemental Figure 8. Analyses of RIDD target genes.** (**a**) The abundance of adult head mRNAs encoding the indicated RIDD target genes was determined by quantitative RT-PCR for GMR-Gal4>+, GMR-Gal4>*Ire1*-Ri, GMR-Gal4>IRE1;*Ire1*-Ri flies. Data are shown as mean ± s.e.m. (n=30 flies/genotype from at least two independent experiments). **P<*0.05, ***P<*0.01, ****P<*0.001 by one-way ANOVA. (**b**) Representative light microscopy of external eyes from adult GMR-Gal4>IRE1, GMR-Gal4>*Fatp*-Ri, GMR-Gal4>*Cds*-Ri, and GMR-Gal4>*Indy*-Ri flies versus GMR-Gal4>+ flies (n=5-8 flies/genotype). Scale bar represents 100 µm.

**Supplemental Figure 9. Knockdown of *Ire1* or *Atg7* expression in dopaminergic neurons does not affect lifespan, locomotive activity or neuronal viability.** (**a**) Lifespan of Ddc-Gal4>+, Ddc-Gal4>*Ire1*-Ri, or Ddc-Gal4>*Atg7*-Ri flies (n=90 flies/genotype). (**b**) Climbing ability of the indicated lines at 1, 3 or 5 weeks of age. Relative activities are shown as mean ± s.e.m. (n=90 flies/genotype; two independent experiments). (**c**) Z-stack confocal micrography of whole brains stained with anti-TH antibody. Shown are representative images of the indicated lines at 1 day or 40 days of age. TH-positive neurons of the indicated clusters were quantified and are shown as mean ± s.e.m. (n=5 flies/genotype; two independent experiments). Scale bar represents 100 µm.
